# The quality of instruments to assess the process of shared decision making: A systematic review

**DOI:** 10.1371/journal.pone.0191747

**Published:** 2018-02-15

**Authors:** Fania R. Gärtner, Hanna Bomhof-Roordink, Ian P. Smith, Isabelle Scholl, Anne M. Stiggelbout, Arwen H. Pieterse

**Affiliations:** 1 Department of Medical Decision Making, Leiden University Medical Centre, Leiden, the Netherlands; 2 Department of Medical Psychology, University Medical Center Hamburg-Eppendorf, Hamburg, Germany; 3 The Dartmouth Institute for Health Policy and Clinical Practice, Lebanon, NH, United States of America; TNO, NETHERLANDS

## Abstract

**Objective:**

To inventory instruments assessing the process of shared decision making and appraise their measurement quality, taking into account the methodological quality of their validation studies.

**Methods:**

In a systematic review we searched seven databases (PubMed, Embase, Emcare, Cochrane, PsycINFO, Web of Science, Academic Search Premier) for studies investigating instruments measuring the process of shared decision making. Per identified instrument, we assessed the level of evidence separately for 10 measurement properties following a three-step procedure: 1) appraisal of the methodological quality using the COnsensus-based Standards for the selection of health status Measurement INstruments (COSMIN) checklist, 2) appraisal of the psychometric quality of the measurement property using three possible quality scores, 3) best-evidence synthesis based on the number of studies, their methodological and psychometrical quality, and the direction and consistency of the results. The study protocol was registered at PROSPERO: CRD42015023397.

**Results:**

We included 51 articles describing the development and/or evaluation of 40 shared decision-making process instruments: 16 patient questionnaires, 4 provider questionnaires, 18 coding schemes and 2 instruments measuring multiple perspectives. There is an overall lack of evidence for their measurement quality, either because validation is missing or methods are poor. The best-evidence synthesis indicated positive results for a major part of instruments for content validity (50%) and structural validity (53%) if these were evaluated, but negative results for a major part of instruments when inter-rater reliability (47%) and hypotheses testing (59%) were evaluated.

**Conclusions:**

Due to the lack of evidence on measurement quality, the choice for the most appropriate instrument can best be based on the instrument’s content and characteristics such as the perspective that they assess. We recommend refinement and validation of existing instruments, and the use of COSMIN-guidelines to help guarantee high-quality evaluations.

## 1. Introduction

There is growing recognition that shared decision making (SDM) is imperative as a decision making model in clinical practice when more than one option is medically relevant or when patient preferences vary strongly. Various conceptual models describe what the process of SDM between health care providers and patients entails [1, 2]. Many of these models describe steps that have to be taken as part of SDM. In a recent paper, Stiggelbout and colleagues identify four key steps: “(*1) the professional informs the patient that a decision is to be made and that the patient's opinion is important; (2) the professional explains the options and their pros and cons; (3) the professional and the patient discuss the patient's preferences and the professional supports the patient in deliberation; (4) the professional and patient discuss the patient’s wish to make the decision*, *they make or defer the decision*, *and discuss follow-up*.*”* [2] SDM aims to promote patient autonomy, to limit practice variation, and ensure that treatment decisions reflect patient preferences [1, 3, 4]. Research shows that the occurrence of SDM in routine clinical practice is still limited [5, 6]. Current research agenda focuses on studies on the level of SDM seen in clinical care [5], effects of training and tools for healthcare providers and patients to promote SDM in the clinical practice [7, 8], and the effect of SDM on psychosocial and physical patient outcomes [9–11]. The quality of these studies highly depends on the availability of psychometrically sound instruments to assess the actual realization of SDM. It is notable that the SDM measures used vary greatly with regard to their characteristics, such as the source of the data and the perspective of the scorers (self-report questionnaires based on the experience of patients or providers versus coding schemes applied by independent raters to audio- or video-taped consultations) [12]. These differences can impact research outcomes, as might be the case for a review on the relationship between SDM and patient health outcomes which found that the perspective from which SDM is measured affects the associations found with health outcomes [8]. Furthermore, it is not clear if there are differences in measurement quality between different instruments. To assist researchers in their choice of the most feasible, reliable, and valid SDM measure, and to optimally improve existing instruments, insight into measurement quality of the existing measures is needed.

Previous literature reviews have provided an overview of existing instruments, but have not systematically appraised the quality of the instruments’ measurement properties in a process that accounts for the methodological quality of their validation [12–15] Concerning the instruments’ measurement quality, the existing reviews only presented results on reliability and validity testing in a descriptive manner. None of the previous reviews systematically appraised the quality of the measurement properties of existing instruments, taking into account the methodological quality of their validation studies. In any study, poor methodological quality can bias the results. Consequently, when drawing conclusions on the quality of measurement instruments, one should appraise and correct for the risk of bias arising from the methods applied in the validation studies of the instruments under investigation [16]. Therefore, we aim to perform a systematic literature review that presents an overview of all SDM process instruments and their measurement quality, by answering the following research question: What is the measurement quality of existing instruments measuring the process of SDM, taking into account the methodological quality of the available validation studies?

This systematic review was registered at PROSPERO: CRD42015023397 Available from: **https://www.crd.york.ac.uk/PROSPERO/display_record.php?RecordID=23397**

## 2. Methods

### 2.1 Search strategy

Seven electronic databases (PubMed, Embase, Emcare, Cochrane, PsycINFO, Web of science, Academic Search Premier) were systematically searched for peer-reviewed articles in May 2015 and the search was updated on September 1, 2017. A librarian experienced in systematic searches of academic databases assisted the researchers in developing and performing the search strategy. Our search strategy was developed in line with recommendations and existing search filters specifically developed for systematic reviews, assessing the measurement quality of measurement instruments in the medical field, described by Terwee and colleagues [17]. We combined three search groups with the Boolean operator AND: Group I consisted of search terms presenting the construct of interest, i.e., SDM; group II consisted of search terms for instrument types, such as questionnaire and coding schemes; and group III consisted of search terms for measurement properties. Index terms specific for each database (such as MESH and Major terms in PubMed) were combined with free-text words. We added a fourth search group using the Boolean operator NOT, to exclude specific publication types such as editorials. The complete search strategy is presented in the Appendix. We then reviewed all articles citing the of articles that meet our inclusion criteria to check for additional relevant articles with a publication date prior to October 10, 2017. Furthermore, we contacted a network of SDM researchers via the Shared-l mailing list (Shared-l@shared-l.org; http://www.psych.usyd.edu.au/mailman/listinfo/shared-l) and asked them to inform us of any ongoing studies related to the development or evaluation of instruments measuring the process of SDM.

### 2.2 Selection of eligible articles

The search aimed to include all articles that describe the development or evaluation of instruments that measure the SDM process, which is an assessment of the actual realization of SDM in clinical practice. Articles that evaluate instruments measuring antecedents of SDM (e.g., preferred role in decision making) or SDM outcomes (such as decisional regret) were not included. The inclusion criteria are presented in detail in Table 1. To check eligibly for inclusion, each article retrieved in the search was independently assessed by two members of the research team (MB, HB-R, FG, IPS, IS, AP). In a twofold process, researchers reviewed the titles and abstracts of each article. If these indicated potential inclusion, the full-text of the article was assessed using the inclusion criteria. Disagreements were resolved in consensus between the two reviewers and a third reviewer was consulted if necessary.

**Table 1:** Eligibility criteria.

Inclusion criteria 1. The article had to describe a primary study in which the development or evaluation of one or more instruments occurred. 2. Instruments under investigation: a. were developed with the aim of measuring the process of SDM between a patient (with or without family) or proxy and a healthcare provider; or b. were evaluated in their ability to measure the process of SDM even though they were not originally developed to measure the process of SDM; or c. were developed or evaluated in their ability to measure patient participation in decision making. To guarantee a focus on SDM, these instruments should assess at least one of four key steps of SDM [8, 18, 19]: i. explaining that a decision has to be made, ii. discussing all relevant treatment options and their associated benefits and harms, iii. discussing patients’ ideas, concerns and expectations and supporting patients in the process of deliberation, before reaching a decision, iv. patient involvement in making the final decision. 3. The article had been peer-reviewed. (Not applicable to unpublished work received via the SHARED e-mail list.) 4. The article was written in English, Dutch, or German. Exclusion criteria To guarantee that the instrument under investigation measures a decision making process that includes both the health care provider and the patient, the following two exclusion criteria were applied: 1. Articles investigating instruments that measure inter-professional SDM that does not include the participation of patients. 2. Articles about instruments developed or evaluated for the measurement of SDM about screening. These decisions often rather relate to informed decision making and thus crucially differ from SDM in two aspects: a) the healthcare provider is not necessarily involved in making the decision; b) a decision usually is not needed by a certain time point. No restrictions were held for: 1. The type of measurement instrument (e.g. self-report questionnaire or coding scheme), 2. The healthcare setting in which the instrument was evaluated.

### 2.3 Data extraction

For each included article we extracted data on the methods (setting, healthcare provider sample, patient sample, data collection and coders in case of observer-based data), and results for 10 measurement properties (see Table 2). In case an article describes the evaluation of multiple instruments, the data extraction was performed separately for each instrument under investigation. The extracted data is presented in the online Supporting Information (S1 Table); this data is a summary of the methods and results of the included validation studies and informs the quality appraisals that we performed, as described in section 2.5. For each instrument identified by the included articles we extracted i) the instrument’s measurement aim and construct, ii) the measurement characteristics, i.e., underlying measurement model, number of subscales and items, response scale, and score range, and iii) details on the development process. For each included article, the data was extracted by one and checked by a second project team member (HB-R, FG, IS, ISCH, AP, AS); disagreements between these two were discussed until consensus was reached. In case of doubt a third researcher was consulted. Only information listed in the included article was extracted and considered for assessment, unless the article specifically referred to some other source for this information.

**Table 2:** Definition of measurement properties based on COSMIN [20] and Terwee et al.[21].

**Measurement property**	**Definition**
**I. Reliability**	
** Internal consistency**	The degree to which items in a (sub)scale are intercorrelated, thus measuring the same construct.
** Reliability**	The extent to which subjects can be distinguished from each other, despite measurement errors (relative measurement error).
** Measurement error/ Agreement**	The degree to which the scores on repeated measures are close to each other (absolute measurement error).
**II. Validity**	
** Content validity**	The degree to which the instrument is an adequate reflection of the construct to be measured.
** Construct validity**	
Structural validity	The degree to which the scores of the instrument are an adequate reflection of the dimensionality of the construct to be measured.
Hypotheses testing	The degree to which the scores of the instrument are consistent with hypotheses, based on the assumption that the instrument validly measures the construct to be measured.
Cross-cultural validity	The degree to which the performance of the items on a translated or culturally adapted instrument are an adequate reflection of the performance of the items of the original version of the instrument.
** Criterion validity**	The degree to which the scores of the instrument are an adequate reflection of a ‘gold standard’.
**III. Responsiveness**	
** Responsiveness**	The ability of the instrument to detect changes over time in the construct measured.
** Interpretability**	Interpretability is the degree to which one can assign qualitative meaning- that is, clinical or commonly understood connotations–to an instrument’s quantitative scores or change in scores.

### 2.4 Quality appraisal of measurement properties of SDM instruments

For each instrument, we appraised the quality of ten measurement properties (see Table 2) described in the validation studies in two ways. First, we rated the quality of the methods used to evaluate the measurement properties of an instrument; from here on referred to as the appraisal of methodological quality. Second, we rated the measurement properties based on the results of the validation studies. Data from these two appraisals were combined to provide a best-evidence synthesis of the quality of the measurement properties for each instrument included.

#### 2.4.1 Appraisal of methodological quality

To appraise the methodological quality we used the COnsensus-based Standards for the selection of health status Measurement INstruments (COSMIN) checklist [20, 22, 23]. The COSMIN checklist describes how ten different measurement properties should ideally be evaluated and provides scoring criteria for the methodological quality appraisal. For each measurement property, the quality of the methods used to evaluate it is scored by a number of items (ranging from 4 to 18) on a four-point rating scale: “excellent”, “good”, “fair”, or “poor”. For some items, the lowest response options were “good” or “fair”. The scoring criteria for each category on the rating scale are uniquely defined per item. The overall score per measurement property was determined by taking the lowest item-level score for that specific measurement property. That is, if one item in a property was rated as “poor” then the entire property was rated as “poor”. For instruments following item response theory (IRT), specific IRT criteria were scored, instead of internal consistency and structural validity. There are no COSMIN criteria to appraise methodological quality for the property interpretability. Therefore, for interpretability we only inventoried if two aspects of interpretability were evaluated, i.e., floor and ceiling effects, and minimal important change value. More information on COSMIN and the checklist items can be found on http://COSMIN.nl.

The 10 measurement properties and their definitions based on COSMIN [20] and Terwee et al.[21] are presented in Table 2. Due to variability in the field regarding names used for measurement properties, we classified the measurement properties evaluated in included articles using the terminology and definitions of COSMIN [20] and Terwee et al.[21] (see Table 2) rather than the labels given by the authors of the articles. For example, if authors used the term ‘convergent validity testing’ to designate the testing of hypotheses about the relationship of the instrument under investigation with another existing instrument measuring related constructs, we extracted and evaluated this information using COSMIN criteria for hypotheses testing.

We scored reliability separately for test-retest reliability (applicable to questionnaires only), inter-rater reliability, and intra-rater reliability (the latter two being applicable to coding schemes only). Items about reliability that were not applicable to the inter-rater reliability and intra-rater reliability of coding schemes, were omitted in the rating of the methodological quality of validation studies evaluating coding schemes, i.e., for intra-rater reliability item 7 (*Were patients stable in the interim period on the construct to be measured*?); for inter-rater validity: item 6 (*Was the time interval stated*?), item 7 (*Were patients stable in the interim period on the construct to be measured*?), and item 8 (*Was the time interval appropriate*?).

We applied two modifications to the COSMIN rating. First, we diminished the impact of the item “Was there a description of how missing items were handled?” on the total score for a measurement property. This item is included in the rating of most measurement properties and often received the lowest possible score, a “fair” rating. This score often was the lowest score on the measurement property and would then obscure how the other methodological aspects for that measurement property were rated. We therefore decided to let this item have less impact on the final score by upgrading the total score on a measurement property in case the score on this specific item was the lowest of all scores. E.g., if all items for the measurement property had received “good” or “excellent” rating, and the score on this specific item was a “fair”, the total score was set on “good”, or: if all items had been rated as “excellent” and the score on this specific item was a “fair”, the total score was set at “good”.

Second, we adapted the rating of content validity. The COSMIN checklist requires that for content validity testing, three types of relevance should be assessed, regarding a) the construct to be measured, b) the study population, and c) the purpose of the measurement instrument. These requirements are quite stringent and therefore we have adapted the scoring of these three items as follows: If one or two types of relevance were missing, the concerning items were not scored. The score for items concerning the type of relevance that *was* assessed was downgraded by one score. That is, an excellent score for content validity testing was only possible when two or more types of relevance had been assessed.

#### 2.4.2 Appraisal of the measurement properties

To rate the measurement property of an instrument within a particular study, we used three possible quality scores: a positive rating (labeled +), an inconclusive rating (labeled?), and a negative rating (labeled -). The criteria we used were based on Terwee et al.[21] and Schellingerhout et al. [24, 25] and are presented in Table 3.

**Table 3:** Quality criteria for results on measurement properties based on Terwee et al.[21].

Measurement property	Criteria for appraisal of the results on measurement properties evaluation	
**Internal consistency**	+	Cronbach’s alpha(s) are ≥ 0.70
	?	Not able to score because of unclear or missing information, e.g., the dimensionality is not known or Cronbach’s alpha(s) are not presented.
	-	Criteria for ‘+’ not met.
**Reliability**	+	ICCagreement/weighted Kappa ≥ 0.70 OR ICCconsistency/ICC without approach stated/Pearson’s r ≥ 0.80 OR unweighted kappa/or kappa without approach stated ≥ 0.80
	?	Not able to score because of unclear or missing information, e.g., neither ICC, Kappa, nor Pearson’s r is determined.
	-	Criteria for ‘+’ not met.
**Measurement error/ Agreement**	+	MIC ≥ SDC OR MIC outside the LOA OR convincing arguments that agreement is acceptable
	?	Not able to score because of unclear or missing information, e.g. SEM, SDC not calculated, or MIC not defined.
	-	Criteria for ‘+’ not met.
**Content validity**	+	Target group and/or experts considered all items to be relevant AND considered the item set to be complete.
	?	Not able to score because of unclear or missing information, e.g. no results on item relevance according to experts reported
	-	Criteria for ‘+’ not met.
**Construct validity**		
Structural validity	+	**For exploratory factor analyses:** Factors chosen explain at least 50% of variance OR factors chosen explain less than 50% of variance but the choice is justified by the authors. **For confirmatory factor analyses:** (The goodness of fit indicators fulfil the following requirements: (CFI or TLI or GFI or comparable measure >0.90) AND (RMSEA or SRMR < 0.08)) AND (results confirm models with the original factor structure OR results confirm a model with slight changes if these changes are justified by the authors.
	?	**For exploratory factor analyses:** Not able to score because of unclear or missing information, e.g. explained variance not mentioned. **For confirmatory factor analyses:** Not able to score because of unclear or missing information, e.g., no fit indices are presented
	-	Criteria for ‘+’ not met.
Hypotheses testing	+	(At least 75% of the results are in accordance with the hypotheses AND, if calculated, the correlation with an instrument measuring the same construct is ≥ 0.50) AND correlations with related constructs are higher than with unrelated constructs if calculated.
	?	Not able to score because of unclear or missing information, e.g. no correlations with related construct are calculated
	-	Criteria for ‘+’ not met.
Cross-cultural validity	+	The original factor structure is confirmed AND no important DIF found. If only one of these properties are investigated: either the factor structure is confirmed OR no important DIF found.
	?	Not able to score because of unclear or missing information, e.g. no confirmative factor analyses is performed nor the DIF is investigated.
	-	Criteria for ‘+’ not met
Criterion validity	+	Correlations with chosen gold standard ≥ 0.70, OR AUC ≥ 0.80, OR (specificity AND sensitivity ≥ 80).
	?	Not able to score because of unclear or missing information
	-	Criteria for ‘+’ not met.
**Responsiveness**	+	Correlations of change scores of the target instrument with an instrument measuring the same construct are ≥ 0.40 OR at least 75% of the results are in accordance with the hypotheses OR AUC ≥ 0.70) AND Correlations of change scores of the target instrument with an instrument measuring a related constructs are higher than with unrelated construct if calculated.
	?	Not able to score because of unclear or missing information, e.g. no correlations of change score with related constructs are calculated or no AUC investigated.
	-	Change score correlation with an instrument measuring the same construct < 0.40 OR < 75% of the results are in accordance with the hypotheses OR AUC < 0.70 OR change score correlations with related constructs are lower than with unrelated constructs.
**Interpretability**	*No quality scoring performed*	
**Item response theory (IRT)**	+	At least limited evidence for unidimensionality or positive structural validity AND no evidence for violation of local independence: Rasch: standardized item-person fit residuals between -2.5 and 2.5; OR IRT: residual correlations among the items after controlling for the dominant factor < 0.20 OR Q3's < 0.37 AND no evidence for violation of monotonicity: adequate looking graphs OR item scalability >0.30 AND adequate model fit: Rasch: infit and outfit mean squares ≥ 0.5 and ≤ 1.5 OR Z-standardized values > -2 and <2 OR IRT: G2 >0.01. Optional additional evidence: Adequate targeting; Rasch: adequate person-item threshold distribution; IRT: adequate threshold range. No important DIF for relevant subject characteristics (such as age, gender, education), McFadden's R2 < 0.02
	?	Model fit not reported
	-	Criteria for ‘+’ not met

+ = positive result for a measurement property

? = result of measurement property is unknown

- = negative result for a measurement property

#### 2.4.3 Best-evidence synthesis

As recommended by Terwee et al [16] we determine the overall quality of a particular measurement property of an instrument. We used the approach of Schellingerhout and colleagues [24, 25], in which the results from the different articles are synthesized for each instrument by combining: the appraisal of methodological quality of the studies (see 2.5.1), the appraisal of the measurement property (see 2.5.2), the number of studies assessing the property, and the consistency of the results in case of multiple validation studies. For this overall rating, five levels of evidence were applied: unknown evidence (?), conflicting evidence (+/-), limited (+ or -), moderate (++ or--), and strong evidence (+++ or---). The latter three could point in either a positive or negative direction, which we indicated by respectively using the plus sign and minus sign. The scoring criteria are presented in Table 4.

**Table 4:** Levels of evidence for the best-evidence synthesis.

Level of evidence	Rating	Criteria
Strong	+++ or ---	Consistent findings in multiple studies of good methodological quality OR one study of excellent methodological quality
Moderate	++ or --	Consistent findings in multiple studies of fair methodological quality OR one study of good methodological quality
Limited	+ or -	One study of fair methodological quality
Conflicting	+/-	Conflicting findings
Unknown	?	Only studies of poor methodological quality

A plus sign (+) indicates = positive results for a measurement property evaluation and a minus sign (-) indicates negative results for a measurement property evaluation, e.g., + stands for limited evidence for positive results and --- stands for strong evidence for negative results for a measurement property.

Two members of the research team (HB-R, FG, IPS IS, AP) rated the methodological quality and measurement properties of each article, with discrepancies discussed until consensus was reached. In case of doubt a third team member was consulted. For the methodological quality appraisal, consensus had to be reached on the item-level, not only on the total scores per measurement property rated. One team member performed the best-evidence synthesis (FG) and a second (AP) checked it. Team members who were co-author of an included article were not involved in data extraction and quality appraisals of that article. For instruments consisting of multiple subscales, we performed the quality appraisals of the methods and properties separately for each subscale. To provide an overall score for a measurement property for these instruments, we used the lowest subscale scores as input for the data synthesis.

## 3. Results

### 3.1 Search results

**Fig 1:**
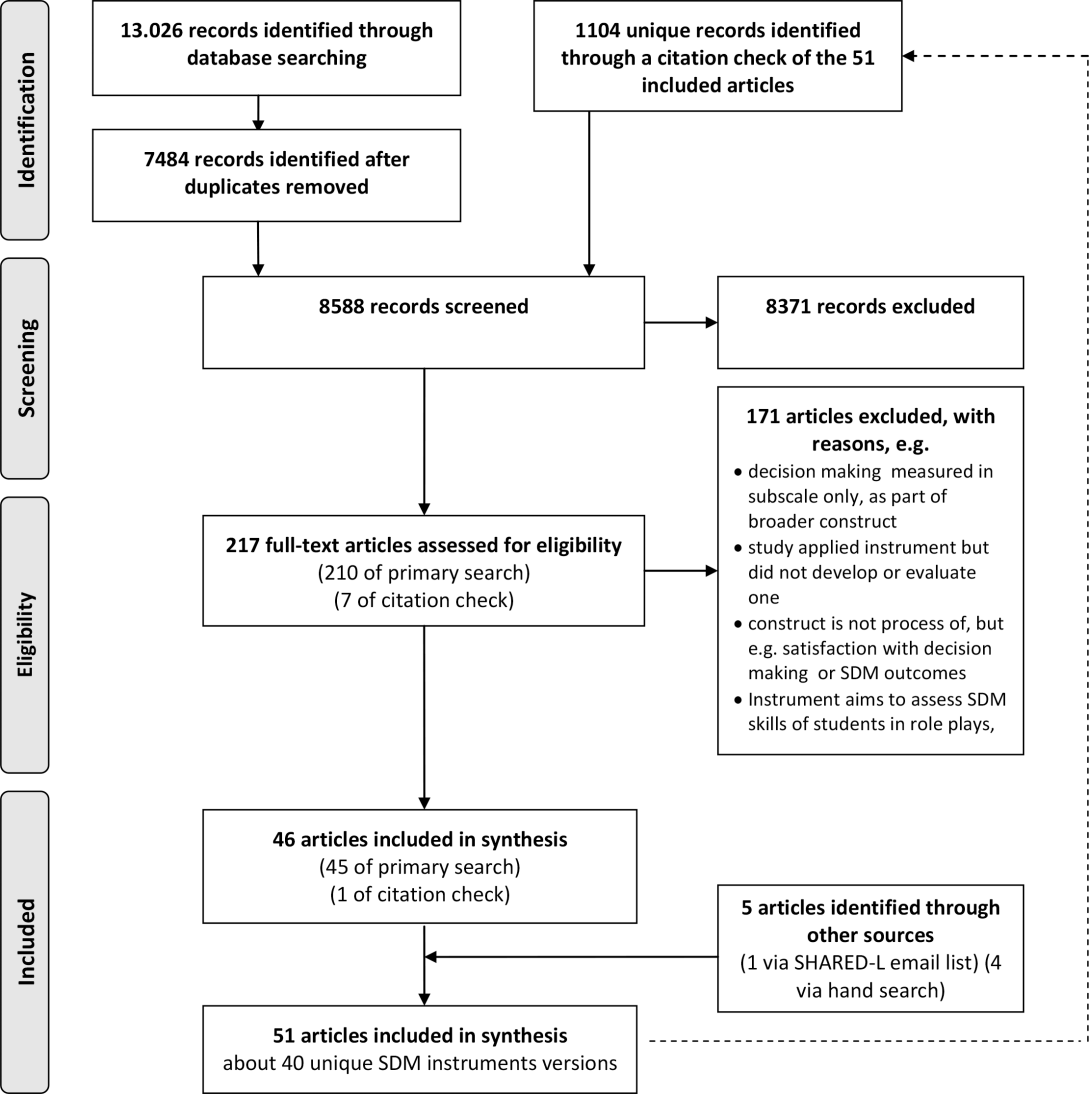
Flow diagram of article selection process.

**Fig 2:**
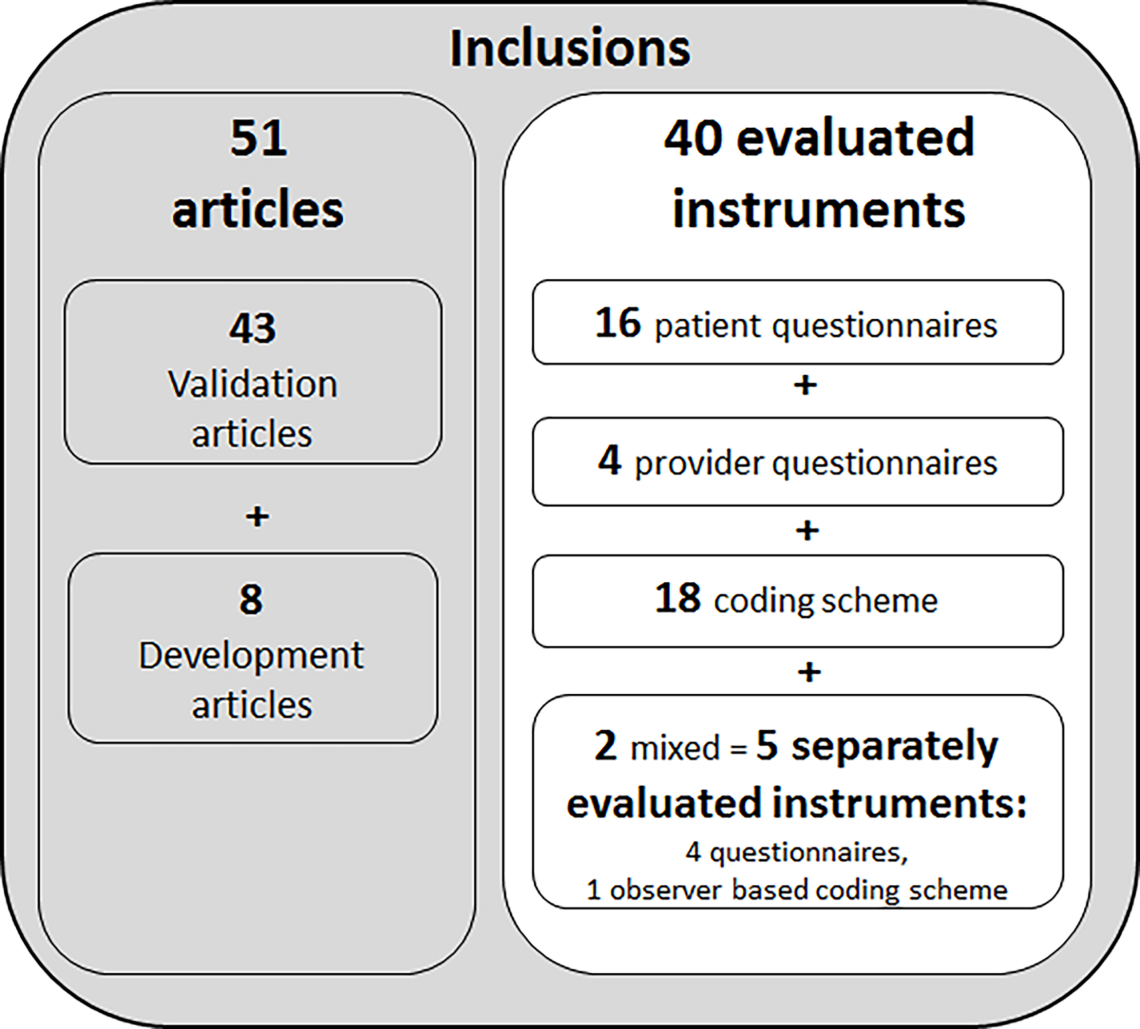
Number of included articles and instrument.

The primary search in seven databases retrieved 13.026 articles, of which, after removing duplicates, 7484 unique hits were screened for inclusion. Another 1104 unique articles were identified by the citation check of all articles that were eligible for inclusion in this systematic review. After title abstract screening, 217 articles were assessed for eligibility based on their full-text. In total, fifty one articles met our inclusion criteria (Fig 1), of which forty-five derived from the primary search, one from the citation check, 4 through the call in the e-mail list of SDM researchers and 1 via hand search. The 51 included articles describe the development and/or evaluation of 40 unique instruments that assess the process of SDM (Fig 2). In total 21 instruments were originally developed versions, 4 were revised versions, and 15 were translated versions. In Table 5, we describe the characteristics of the instruments. Most instruments were observer-based coding schemes (N = 18), followed by patient questionnaires (N = 16) and provider questionnaires (N = 4); two were mixed, including two or more instruments assessing multiple perspectives: the dyadic OPTION, consisting of a patient and a provider questionnaire [26] and the Mappin’SDM, consisting of a patient questionnaire, a provider questionnaire, and a coding scheme [27]. For the quality appraisal of mixed instruments, we rated the measurement quality of mixed instruments separately for each perspective; result in a total number of instruments for which we performed a best evidence synthesis of N = 43. The number of validation studies per instrument varied between zero and four. For most instruments (N = 28), one validation article has been published.

**Table 5:** Characteristics of the instruments measuring the process of SDM regarding the construct and the instruments’ measurement features.

Instrument	1^st^ author, publication year	Perspective	Version	Language	Target setting	Measurement aim	Construct and its definition	Measurement model (formative versus reflective)	Number of Subscales (total number of items) 1. name of subscales 1(# items), 2. Name of subscales 2 (# items),	Response-scale; total score range	Development process a) how construct defined; b) item generation; c) item selection; d) pilot test e) (cultural) adaptation/translation process
**Patient questionnaires**											
**PPC** Patients' preferences for control	Bradley, 1996 [36]	Patient	Original	assumed to be English	Generic	Patient desire for involvement in making medical decisions in general and in 10 scenarios depicting different acute and chronic medical situations	Not reported	Not applicable because exists of 1 item only	1 (1)	7-point scale: 1 = I prefer that my doctor tell me what to do to 7 = I prefer that I make the decision without any information or recommendation from the doctor; not reported	a) Literature; b) by authors (family physician, internist, social worker), based on the literature, clinical scenarios were then reviewed by family physicians (N = 2); c) not reported; d) lay people (N = 12) assessed readability and understanding of items; e) n/a
**CPS post** post Control Preferences Scale (actual role)	Degner, 1997 [37]	Patient	Original	assumed to be English	Generic	Consumer preferences regarding participation in health care decisions (Aim for CPS post not reported)	**Control preferences**: Control preferences is the degree of control an individual wants to assume when decisions are being made about medical treatment (Definition for perceived actual role not reported)	Not applicable because exists of 1 item only	1 (1)	5 role descriptions: A = I prefer to make the decision about which treatment I will receive to E = I prefer to leave all decisions regarding treatment to my doctor, (labels for assessing actual role not reported), two possible procedures: Order of 5 cards with role descriptions (card sort task) or selection of 1 role ("pick one" approach); not reported	Card sort task, preferred role: a) Literature and qualitative work by authors; b) participant observation; c) not reported, d) pilot test 1: Tested in 60 cancer patients and problematic statements revised; pilot test 2: Tested in 30 cancer patients and cartoons added; e) n/a
**FPI** Facilitation of Patient Involvement Scale	Martin, 2001 [28]	Patient	Original	assumed to be English	Generic	Degree to which patients perceive that their provider actively facilitate or encourage them to be involved in their own healthcare	**Facilitating or promoting a patient’s involvement in care**: Facilitating or promoting a patient’s involvement in care entails communicating openly with the patient, giving information, and allowing the patient to express his or her views and opinions	Assumed to be reflective as Cronbach's alpha calculated	1 (9)	6-point scale: 1 = none of the time to 6 = all of the time; not reported	a) Unclear; b) based on literature review; c) expert review (N = 17 research psychologists) of face validity, content overlap, and ambiguity, led to removal and modification of items; d) not reported; e) n/a
**COMRADE** Combined Outcome Measure for Risk communication And treatment Decision making Effectiveness	Edwards, 2003 [38]	Patient	Original	assumed to be English	Generic	Effectiveness of risk communication and treatment decision making in consultations	**Risk communication**: Risk communication is the open two-way exchange of information and opinion about risk, leading to better understanding and better (clinical) management decisions; **Effective decisions**: Effective decisions are decisions that are informed, consistent with personal values and acted upon	Assumed to be reflective as Cronbach's alpha calculated	2 (20); 1. Risk communication (10), 2. Confidence in decision (10)	Unclear; total score range for each subscale: 0–100	a) Literature; b) existing instruments identified through systematic literature review, semi-structured focus group interviews with patients (N = 49), and interviews with general practitioners (N = 6); c) in an iterative process the (group) interview data plus written feedback on face validity, simplicity and ambiguity of items led to revision and elimination of items; d) 72 patients at five general practices completed the questionnaire after consultation with a doctor and 20 of these patients were interviewed on item clarity; e) n/a
**SDM-Q** Shared Decision-Making Questionnaire	Simon, 2006 [39]	Patient	Original	German	Generic	SDM process in clinical encounters	**SDM**: An SDM process consists of the following nine sequential steps: 1. Disclosure that a decision needs to be made, 2. Formulation of equality of partners, 3. Equipoise statement, 4. Informing on the benefits and risks of options, 5. Investigation of patient’s understanding and expectations, 6. Identification of preferences, 7. Negotiation, 8. Shared decision, 9. Arrangement of follow-up	IRT	1 (11)	4-point scale: 0 = strongly disagree to 4 = strongly agree; not reported	a) Literature and nominal group technique-based discussions; b) Delphi method; c) pilot testing and item fit analysis; d) piloted in readability tests with patients as well as experts in questionnaire development; e) n/a
**SDM-Q-9** 9-item Shared Decision-Making Questionnaire	Kriston, 2010 [29]	Patient	Revision	German	Generic	SDM process in clinical encounters	**SDM**: SDM is as an interactive process in which both parties (patient and provider) are equally and actively involved and share information in order to reach an agreement, for which they are jointly responsible	Assumed to be reflective based on comment in discussion	1 (9)	6-point scale: 0 = completely disagree to 5 = completely agree; 0–45, rescaled range: 0–100	a) Literature review and previous SDM-Q instrument; b) author-generated based on research experience; c) equally weighted criteria: face validity by researchers (N = 2), and by acceptance, discrimination, difficulty in primary care sample (N = 1163); d) not reported; e) n/a
**SDM-Q-9** (Spanish)	De las Cuevas, 2014 [32]	Patient	Translation	Spanish	Generic	SDM process in clinical encounters	**SDM**: SDM is an interactive process of clinical decision making that ensures that both patient and physician are equally and actively involved and share information to reach an agreement, for which they are jointly responsible	Reflective	1 (9)	6-point scale: 0 = completely disagree to 5 = completely agree; not reported	a) Literature; b-d) n/a; e) following 5 steps according to guideline, including multiple forward and multiple backward translations and consensus discussions with translators and authors of original instrument; rating of content validity and understandability and semantic and content equivalence of the German and Spanish versions by independent experts (primary care physicians, psychiatrists, psychologists) (N = 5). Pre-test of the final version in adult patients (N = 12) at one of two primary care health centres. No further modifications were necessary after this pre-test.
**SDM-Q-9** (Dutch)	Rodenburg, 2015 [33]		Translation	Dutch	Generic	SDM process during a consultation	**SDM**: In partnership with their providers, patients are encouraged to consider the likely harms and benefits of available treatment options, communicate their preferences, and select the option that best fits these	Assumed to be reflective as Cronbach's alpha calculated	1 (9)	6-point scale: 0 = completely disagree to 5 = completely agree; 0–45, rescaled range: 0–100	a) Literature; b-d) n/a; e) multiple forward-backward translations of the original German version by two native Dutch and two native German speakers, comparison and discrepancy discussion in consensus meeting with four team members, including author of original German version; final version presented to clinicians for their opinion on wording (N not reported)
**SDM-Q-9 Psy** (Hebrew)	Zisman-Ilani, 2016 [34]	Patient	Translation	Hebrew	Psychia-try	Decision making processes and SDM practice in real-time consultations with people with serious mental illness who are currently hospitalized in psychiatric hospitals	**SDM**: SDM is an interactive process in which patient and provider are equally and actively involved and share information to reach an agreement about treatment for which they are jointly responsible	Assumed to be reflective as Cronbach's alpha calculated	1 (9)	6-point scale: 0 = completely disagree to 5 = completely agree; not reported	a) Literature; b-d) n/a; e) authors translated and made a few contextual and lingual adaptations based on the guidelines for cross-cultural adaptation by Beaton et al 2000, Spine 25, 3186–3191
**SDM-Q-9** (English)	Alvarez, 2017 [30]	Patient	Translation	English	Generic	To evaluate patient-reported SDM from a patient-provider visit based on the patient’s perception.	**SDM**: SDM is” a form of patient-provider communication where both parties bring expertise to the process and work in partnership to make a decision” (Duncan, Best, & Hagen, 2010).	Assumed to be reflective as Cronbach's alpha calculated	1 (9)	6-point scale: 0 = completely disagree to 5 = completely agree; 0–45, rescaled range: 0–100	a-d) n/a; e) translated version used
**CollaboRATE**	Elwyn, 2013 [40]	Patient	Original	English	Generic	Extent of SDM in clinical encounters	**SDM**: SDM consists of three core elements: 1. Provision of information or explanation to the patient about relevant health issues or treatment options, 2. Elicitation of the patient’s preferences related to the health issues or treatment options, 3. Preference integration	Formative	1 (3)	Two possible versions: a) CollaboRATE-10: 10-point scale: 1 = no effort was made to 10 = every effort was made; 0–100; b) CollaboRATE-5: 5-point scale: 1 = no effort was made to 4 = every effort was made; 0–12	a) Adapted from literature; b) generated based on construct definition by authors, c) items refined through cognitive interviews; d) 30 participants completed questionnaire; e) n/a
**CollaboRATE** (Swedish)	Rosenberg, 2017 [41]	Patient	Translation	Swedish	Generic	Shared decision making	Not provided	Assumed to be reflective as cronbach’s alpha is calculated	1 (3)	5-point scale: 0 = no effort was made to 4 = every effort was made, 0–12	b-d) n/a; e) First permission to translate was obtained by developers. Second, independent translation by 2 researchers, native Swedish speakers fluent in English, independently translated the instrument into Swedish. Third, retranslation into English by a third researcher, native English speaker also fluent in Swedish, and with no previous knowledge of the original instruments. Fourth, possible transcultural differences between the original and the translated versions were discussed in the research team with the purpose of making the instrument culturally equivalent in order to promote a sound content validity.
**SMDMQ** Taiwan Shared Medical Decision Making Questionnaire	Chang, 2014[42, 43] *	Patient	Original	Taiwanese	Generic	Shared medical decision making process	**Shared medical decision making**: Four components define the shared medical decision making process: 1. Patient Autonomy, 2. Control preference, 3. Patients’ perceived involvement, 4. Risk information communication	IRT	1 (15)	Not reported; 0–15	a) Literature review; b) author generated; c) original 25 items were reduced to 16 based on experts' opinion (N = 12) on content validity and relevance; 1 further item was removed based on Rasch analyses; d) not reported; e) n/a
**SDM Process Score**	Fowler, 2016, in progress [44]	Patient	Original	assumed to be English	Generic	Quality of decision making at a clinical practice or site	**SDM**: In SDM, patients are faced with potential medical tests or treatments for which there are reasonable options, and they should be informed about those options, including the known pros and cons, and should have a voice in making the decisions	Unclear	1 (4)	Items 1–2: 4-point scale: 0 = not at all to 3 = a lot, dichotomized into 0 = not at all or a little to 1 = some or a lot, items 3–4 dichotomous: 0 = no, 1 = yes; 0–4	a) Literature; b) based on previous questionnaire; c) not reported; d) cognitive testing with patients for relevance and clarity of items; e) n/a
**MADM** Mother's Autonomy in Decision Making scale	Vedam, 2017 [45]	Patient	Original	English	Primary maternity care	Women’s autonomy and role in decision making during maternity care	**SDM:** no definition given	Assumed to be reflective as Cronbach's alpha calculated and based on items	1 (7)	6-point scale: 1 = completely disagree to 6 = completely agree; 6–42	a) Literature review; b) items from validated SDM instrument (SDM-Q_9) adapted to maternity setting and new items developed based on feedback from community consultation; c) Expert review and community consultation; d) questionnaire “pilot tested with several women from target population” and revised to improve clarity and logic; e) not applicable
**Dyadic OPTION** (Swedish)	Rosenberg, 2017 [41]	Patient	Translation	Swedish	Generic	perceived patient involvement in shared decision making with the purpose of accessing the dual perspective while using identical items and construct for the patient and the provider version of the questionnaire	Not provided	Assumed to be reflective as cronbach’s alpha is calculated, but original scale was formative	1 (12)	4-point scale: 1 = strongly disagree to 4 = strongly agree; 12–48	a-d) n/a; e) First permission to translate was obtained by developers. Second, independent translation by 2 researchers, native Swedish speakers fluent in English, independently translated the instrument into Swedish. Thrid, retranslation into English by a third researcher, native English speaker also fluent in Swdish, and with no previous knowledge of the original instruments. Fourth, possible transcultural differences between the original and the translated versions were discussed in the research team with the purpose of making the instrument culturally equivalent in order to promote a sound content validity.
**Provider questionnaires**											
**SDM-Q-Doc** Shared Decision Making Questionnaire—provider version	Scholl, 2012 [35]	Provider	Original	German	Generic	Providers’ perspective of the SDM process	**SDM**: Nine practical steps of the SDM process are: 1. Disclosure that decision needs to be made, 2. Formulation of equality of partners, 3. Equipoise statement, 4. Informing on the benefits and risks of options, 5. Investigation of patient’s understanding and expectations, 6. Identification of preferences, 7. Negotiation, 8. Shared decision, 9. Arrangement of follow-up	Assumed to be reflective as Cronbach's alpha calculated	1 (9)	6-point scale: 0 = completely disagree to 5 = completely agree; 0–45, rescaled range 0–100	a) Literature; b, c) adapted existing questionnaire to assess patient perception of SDM (SDM-Q-9); d) completion rates of providers were used as indicator of acceptance: item level completion rate range 94–95%; e) n/a
**SDM-Q-Doc** (Persian)	Ebrahimi, 2014 [46]	Provider	Translation	Persian	Generic	Providers' point of view on SDM	**SDM**: SDM is presenting information for patients to involve them in finalizing the suitable treatment option	Assumed to be reflective as Cronbach's alpha calculated	1 (9)	6-point scale: 0 = completely disagree to 5 = completely agree; 0–45	a) Literature; b-d) n/a; e) Translated from English to Persian by two bilingual experts (1 physician, 1 researcher aware of research objectives); back-translation by a native English speaker (fluent in Persian; unaware of research aims); back-translation was sent for content check to original authors and their recommendations were considered
**SDM-Q-Doc** (Dutch)	Rodenburg, 2015 [33]	Provider	Translation	Dutch	Generic	SDM process during a consultation	**SDM**: In partnership with their clinicians, patients are encouraged to consider the likely harms and benefits of available treatment options, communicate their preferences, and select the option that best fits these	Assumed to be reflective as Cronbach's alpha calculated	1 (9)	6-point scale: 0 = completely disagree to 5 = completely agree; 0–45, rescaled range: 0–100	a) Literature; b-d) n/a; e) multiple forward-backward translations of the original German version by two native Dutch and two native German speakers, comparison and discrepancy discussion in consensus meeting with four team members, including author of original German version; final version presented to clinicians for their opinion on wording (N not reported)
**SDM-Q-Doc** Shared Decision-Making Questionnaire–Physician version	Calderon, 2017 [47]	Provider	Translation	Spanish	Generic	Physicians’ perspectives on SDM processes	Not reported	Assumed to be reflective as factor analysis is performed	1 (9)	6-point scale: 0 = completely disagree to 5 = completely agree; 0–45	a) Literature; b-d) n/a; e) using guidelines for cross-cultural adaptation of self-reported measures; two independent bilingual translators (English, Spanish) translated the English version (a translation of the original German version) into Spanish. Translators reached consensus on the translation of words, phrases and items. Four independent physicians and psychologist rated understandability, translation equivalences and content validity. Another two bilingual translators who were blind to the original English version back translated the revised Spanish version; study directors compared and synthesized the back-translation with the original English questionnaire, and determined the final version. The final version was pre-tested in 34 adult patients no modifications were necessary.
**Observer based coding schemes**											
**IDM** Elements of Informed Decision Making	Braddock, 1999 [48]	Observer	Original	English	Generic	Characterize the completeness of informed decision making during consultations as a function of the complexity of the decision	**Informed decision making**: Informed decision making is a meaningful dialogue between provider and patient	Unclear	1 (7)	Frequencies for two scores: a) if item is required (yes/no), b) if item is present (yes/no); not applicable	a) Literature review and professional consensus; b) earlier work of author and iterative group techniques among providers and laypersons to define completeness for basic, intermediate and complex decisions, and to determine complexity of specific kinds of decisions; c) not reported; d) not reported; e) n/a
**DSAT** Decision Support Analysis Tool	Guimond, 2003 [49]	Observer	Original	assumed to be English	Generic	Providers' use of decision support and related communication skills during clincial encounters	**SDM**: In an SDM situation, patients’ and practitioners’ active cognitive and affective participation is imperative for the success of the interaction. Providers actively elicit patients’ points of view, help them to express themselves openly, and ask questions about issues that affect decision making	Unclear	2 Parts: Part 1, 6 categories of decision support skills (22): 1. Discuss decision making status (5), 2. Discuss knowledge/information (5), 3. Discuss values (4), 4. Discuss support (3), 5. Discuss commitment to act (1), 6. Discuss learning for future decisions (3) and Behaviour not classified (1). Part 2, 4 categories of communication skills (14): 1. Managing the encounter (4), 2. Listening (5), 3. Questioning (2), 4. Sending messages (2) and Behaviour not classified (1)	Frequency of behaviour; not reported	a) Literature and theoretical models; b) existing instrument; c) a panel of researchers, clinicians, and specialists in decision support and communication revised and re-classified the existing instrument; d) not reported; e) n/a
**DSAT-10** Brief Decision Support Analysis Tool	Stacey, 2008 [50]	Observer	Revision	English	Generic	Decision support skills	**Decision support**: Decision support is preparing clients for decision making by providing tailored information, clarifying values, and enhancing self-help skills in decision-making and implementation	Unclear	1 (5 elements encompassing 10 assessment criteria)	dichotomous: present vs. absent; 0–10	a) Based on the Ottowa Decision Support Framework; b) used items from existing DSAT instrument; c) changed and removed items, simplified scoring procedure; d) five coders trained on original DSAT instrument coded encounters between standardized patients and experienced call centre nurses, their findings were discussed and the DSAT-10 was adjusted based on findings; e) n/a
**OPTION** Observing Patient Involvement scale	Elwyn, 2003 [51]	Observer	Original	English	Generic	Extent to which providers involve patient in the decision making process during a consultation	**Patients' engagement in decisions by providers**: Competences of providers to engage patients in decisions: 1. Problem definition (and agreement), 2. Explaining that legitimate choices exist in many clinical situations (i.e., professional “equipoise”), 3. Portraying options and communicating risk about a wide range of issues, 4. Conducting the decision process or its deferment	Assumed to be reflective as Cronbach's alpha calculated	1 (12)	5-point scale: 0 = strongly agree to 4 = strongly disagree; scale range: 0–100	a) Literature review and assessment of clinical practice; b) based on a theoretical framework defining clinical competences of patient involvement in decision making in clinical consultations, developed based on previous instruments review, appraisal of existing research, and qualitative studies with clinicians and patients; c) iterative pilot study with three cycles over a 12 month-period using simulated consultations (N = 6), with GP informants (N = 5) and one non-clinical rater (N = 1); d) non-clinical raters (N = 2) rated audiotaped consultations (N = 7) and gave feedback on feasibilty and acceptability of instrument; e) n/a
**OPTION** (revised)	Elwyn, 2005 [52]	Observer	Revision	English	Generic	Extent to which clinicians involve patients in decision making processes	**Involving patients in decision making**: The process of involving patients in decision making is constituted of clinicians involving patients in the process of understanding the nature of the problem, understanding that there are uncertainties and different likelihoods of harms and benefits, and finally that the patient, if they wish, can influence influence the decision itself	Assumed to be reflective as Cronbach's alpha calculated	1 (12)	5-point scale: 0 = behaviour is not observed to 4 = behaviour is observed and executed to a high standard; 0–100	a) Unclear; b-d) n/a; e) existing coding scheme for which labels of response categories were revised based on user feedback with a shift from an attitudinal to a magnitude-based scale
**OPTION** (Italian)	Goss, 2007 [53]	Observer	Translation	Italian	Generic	Extent to which providers involve patients in decisions	Not reported	Assumed to be reflective as Cronbach's alpha calculated	1 (12)	5-point scale: 0 = behaviour is not observed to 4 = behaviour is observed and executed to a high standard; 0–48, rescaled range: 0–100	a) Missing; b-d) n/a; e) translation of the original English version into Italian by two native Italian speakers and compared to reach consensus. This version was checked for language fluency by a teacher of Italian, was then back-translated into English and compared to the original version by a native English speaker. Subsequently an expert panel reached agreement on a final version. After training of the coders, they added more specific criteria definitions for some items to assist in the interpretation of the items; e) n/a
**OPTION** (revised) (German)	Hirsch, 2011 [54]	Observer	Translation	German	Generic	Extent to which providers involve patients in decisions	Not reported	Assumed to be reflective as Cronbach's alpha calculated	1 (12)	5-point scale: 0 = behavior is not observed to 4 = behavior is observed and executed to a high standard; not reported	a) Not reported; b-d) n/a; e) authors refer to other publication describing 4-stage translation process
**OPTION (revised and modified)** (German)	Keller, 2013 [55]	Observer	Translation	German	Generic	Extent to which providers involve patients in decisions and active involvement of patients	Not reported	Assumed to be reflective as Cronbach's alpha calculated	1 (12)	5-point scale: 0 = not observed to 4 = active involvement of patient is observed (in earlier version, this was: 'high standard'); 0–48	a) Not reported; b-d) n/a; e) existing scale (german OPTION), the label of response category four was modified
**OPTION**^**12**^ (Dutch)	Stubenrouch, 2016 [56]	Observer	Translation	Dutch	Generic	Extent to which healthcare providers involve patients in decision-making	**SDM**: SDM is the process in which both healthcare providers and patients participate to make decisions about their health management strategies, using the best available evidence	Unclear	1 (12)	5-point scale: 0 = no effort to 4 = exemplary effort; 0–60, rescaled range: 0–100	a) Literature; b-d) n/a; e) Dutch version was already available, but after 2 trained coders applied the instrument, the manual was refined to include more extended descriptions of scoring levels
**Observer OPTION**^**5 item**^	Elwyn, 2013 [57]	Observer	Revision	English	Generic	Essential requirements of SDM when providers make an effort to involve patients in decisions	**SDM**: SDM is composed of justifying deliberative work, followed by the steps of describing options, information exchange, preference elicitation, and preference integration	Formative	1 (5)	5-point scale: 0 = no effort to 4 = exemplary effort; 0–100	a) Literature; b-d) n/a; e) items selected from pre-existing shared decision making instrument (Observer OPTION^12^), items selection based on analysis of SDM models and response patterns with OPTION^12^
**OPTION**^**5**^ (Dutch)	Stubenrouch, 2016 [56]	Observer	Translation	Dutch	Generic	Extent to which healthcare providers involve patients in decision-making	**SDM**: SDM is the process in which both healthcare providers and patients participate to make decisions about their health management strategies, using the best available evidence.	Assumed to be formative as is stated for the original version of this instrument	1 (5)	5-point scale: 0 = no effort to 4 = exemplary effort; 0–20, rescaled range: 0–100	a) Literature; b-d) n/a; e) 4 members of the research team, who are native Dutch speakers, translated the original English items independently. All four Dutch translations were back translated by an English speaker with fluent command of the Dutch language. The Dutch versions were revised until agreement was reached. Subsequently, after 2 trained coders applied the instrument, the manual was refined to include more extended descriptions of scoring levels
**RPAD** Rochester Participatory Decision-Making Scale	Shields, 2005 [58]	Observer	Original	assumed to be English	Generic	Provider behaviours that encourage participatory decision making	**Participatory decision making**: Participatory decision making consists of 2 processes: expert problem solving and decision making. *Problem solving* is the province of providers whose expertise informs their judgment to determine treatment options. *Decision making* involves patients working with the provider to determine which treatment options best satisfy the patient’s preferences	Unclear	1 (9)	3-point scale: 0 = no evidence to 1 = description of optimal provider behaviour; 0–9	a) Literature; b) incorporated items suggested in the literature that indicate physician behaviour that encourages patient participation in decision making; c,d) original scale was pilot tested on 10 audio recordings with items that were never coded (N = 5) being discarded, 5 new items were added by authors to complete set; e) n/a
**DAS-O** Decision Analysis System for Oncology	Brown, 2010 [59]	Observer	Original	assumed to be English	Oncology	Quality of key aspects of SDM during oncology consultations in which treatment options, including clinical trials are discussed	**SDM**: Major evaluation criteria for judging adequacy of SDM from provider and patient perspective: 1. Patient understanding of information and the evidence underpinning the treatment choice, 2. Doctor tailoring of information and involvement to the needs of the patient and facilitation of patient decision making by balancing different options and clarifying values, 3. Patient adjustment to and satisfaction with various aspects of the decision making process and the ultimate decision	Unclear	5 (70); 1. Establishing the physician-patient team (22), 2. Following a consultation pathway (13) 3. Providing information about standard treatments and clinical trials (20), 4. Promoting clarity (7), 5. Avoiding coercion (8)	3-point scale: 0 = absent, 1 = basic, 2 = extended; 0–140	a) Literature review; b) qualitative analyses of audiotaped general oncology consultations (N = 26) performed by expert panel from diverse disciplines (ethics, cancer medicine, psycho-oncology, linguistics) following constant comparison method and Systemic Functional Linguistic Approach; c) expert consensus; d) pilot test: authors refer to other publication
**SDM Scale** Shared Decision-Making Scale	Singh, 2010 [60]	Observer	Original	assumed to be English	Oncology	SDM behaviours used by cancer specialists in their consultations	**SDM**: In an SDM model, the patient is given information regarding their disease and possible treatments and is a participant along with the provider in medical decision making	Assumed to be reflective as Cronbach's alpha is calculated	3 (18); 1. Treatment (7), 2. Evidence (8), 3. Patient challenges (3)	2-point scale: 0 = absent, 1 = present or non-applicable; total score: 0–18, subscale 0–10	a) Literature review; b,c) based on literature (including another coding system) a list with key themes and provider behaviours in a consultation was made and reviewed by a team of medical oncologists, oncology nurses and health psychologists; d) coding system created by the team was applied to consultations (N = 5), reviewed, and appropriate adjustments were made; e) n/a
**PES** Parental Engagement Scale	Kearney, 2011 [61]	Observer	Original	no items yet available	Pediatric palliative care	Parental engagement in decision making and planning for seriously ill children during pediatric palliative care consultations	**Parental engagement**: Parental engagement is a psychobehavioral construct that denotes not just parental presence but effective participation	Unclear	3 (not reported); 1. Information-centered dialogue, 2. Insightful participation, 3. Achievement of a collaboratively agreed-upon plan	Not reported;? -9 (lowest score not reported)	a) Literature review and deductive conceptual reasoning; b,c) first: analysis of consultation narratives with content analyses approach, second: integrative process of construct refinement by two researchers based on an iterative process of grouping categories and identifying observable indicators of behaviour for each category; expert content validity checks with clinical experts in paediatric palliative care (N = 3), no results reported; d) actual scoring by two researchers, process not further described; e) n/a
**DEEP-SDM** Detail of Essential Elements and Participants in Shared Decision Making	Clayman, 2012 [62]	Observer	Original	English	Generic	Essential elements of shared decision making	**SDM**: SDM is based on the premise that patients should be involved to the extent that they wish, and their values and preferences are crucial to deciding the ‘‘right” course of action	Unclear	1 (13)	Frequencies, except for the item "Degree of decision sharing: 9-point scale: 1 = physician-led decision to 9 = patient-led decision; not applicable	a) Literature; b) revised previous patient choice instrument and added components; c) not reported; d) two coders independently applied coding scheme to video-recorded consultations of two samples (Sample 1, early stage breast cancer, N = 9; Sample 2, metastatic breast cancer patients, N = 20), modifications and additions were made to the codebook and the examples within definitions to be more inclusive; e) n/a
**Shared decision making rating**	Salyers, 2012 [63]	Observer	Original	assumed to be English	Psychiatry	Level of shared decision making in psychiatric visits	**SDM**: SDM is a collaborative process between a provider and a consumer of health services that entails sharing information and perspectives, and coming to an agreement on a treatment plan	Unclear	1 (9)	3-point scale: 0 = absent to 2 = complete; 0–18	a) Literature; b) an existing coding scheme (Elements of Informed Decision Making Scale) was adapted based on iterative process of individual coding and consensus discussions, a code to one element was added and ratings were added to i) assess who initiated each element to better identify consumer activity and ii) to classify the level of agreement about decision between provider and consumer; c) not reported; d) pilot phase in which inter-rater reliability was 80% for initial coding, 100% after conferencing; e) n/a
**Mappin'SDM**_**norge**_ (Norge)	Kienlin, 2016 (epub ahead of print) [64]	Observer	Translation	Norwegian	Generic	Patient involvement	Not reported	Unclear	3 (11 items each)	5-point scale: 0 = behaviour not observed to 4 = behaviour observed to an excellent standard; not reported	a) Not reported; b-d) n/a; e) pre-existing instrument (MAPPIN'SDM); forward-backward translation, refinement and consensus in research panel including author of original instrument
**Mixed instruments**											
**Dyadic OPTION** (including two questionnaires Dyadic OPTION^Patient^ and Dyadic OPTION^Clinician^)	Melbourne, 2010 [65]	Patient, Provider	Original	English	Generic	Extent to which patients have been involved in (shared) decision making	**Participation in decision making**: Participation in decision making, in particular where attempts are made to share decisions, requires both parties to address the issues of decisional equipoise, compare the features of options and achieve consensus about the best actions	Unclear	1 (12)	4-point scale: strongly agree to strongly disagree; 0–100 (not specified how to calculate)	a) Literature; b,c) observer OPTION adapted for use as questionnaire and cognitive debriefing interviews; d) three rounds of cognitive debriefing interviews, each round consisted of N = 9 participants: N = 3 general practitioners and N = 6 members of the general public, total N = 18. Changes were made after each round; e) n/a
**MAPPIN'SDM Inventory** (including a patient questionnaire, a doctor questionnaire and a coding scheme) (with the possibility to calculate a compound measure, called the SDM Meeting its concept's Assumptions (SDMmass))	Kasper, 2012 [27]	Patient, provider, observer	Original	German	Generic	Interrelations of SDM indicators administered from different perspectives (doctor, patient, observer); (For the SDMmass: Integrative compound measure of SDM)	**Involvement in terms of behaviour:** behaviors attempting to involve the two parties in the decision-making process, i.e., efforts undertaken by doctor or patient to make the particular SDM issue explicit (and by doing so involve each other in the communication). **Result or extent of actual involvement achieved**: perceived (communication) result in terms of SDM, i.e., did the patient or provider feel involved in the communication during the consultation. **SDM:** two way exchange of information within provider-patient dyad involved in decision making.	Assumed to be reflective as Cronbach's alpha calculated	3 observer scales (15 items each): 1. Observer’s perspectives on doctor’s SDM behavior (Obs_doctor_) (15), 2. Observer’s perspectives on patient’s SDM behavior (Obs_patient_) (15), 3. Observer’s perspectives on both parties SDM behaviour (Obs_dyad_) (15); 4 questionnaire scales (15): 1. Doctor’s perspective on SDM behavior (Qdoc_dyad(b)_) (15), 2. Doctor’s perception of SDM result (Qdoc_dyad(r)_) (15), 3. Patient’s perspective on SDM behavior (Qpat_dyad(b)_) (15), 4. Patient’s perception of SDM result (Qpat_dyad(r)_) (15)	Questionnaires: 5-point scale: 0 = not at all to 4 = absolutely true, coding system: 5-point scale: 0 = poor performance to 4 = excellent performance; not reported (scale range of SDMmass: 0 (no SDM)-1 (perfect SDM)	a) Literature review; b) observer scale: OPTION-12 instrument revised and items added by authors to address identified gaps to create a provider instrument, the wording of this instrument was then changed to apply to patient or dyad; questionnaires based on observer instrument; c) n/a; d) questionnaire piloted with physicians (N = 10) and patients (N = 10) resulting in item rewording and addition of explanations; e) n/a

*Reference [42] and reference [43] both presents results of the development and validation for the SMDMQ (Taiwanese), however the results presented seem the exact same in both articles, reference [43] was therefore left out in the data extraction and analysis and also not included in the number of included articles.

### 3.2 Best-evidence synthesis

In Table 6, we present the best-evidence synthesis for each measurement property *per instrument*, (N = 43). For seven instruments (all of which questionnaires), moderate or strong positive evidence was found for at least one type of reliability (internal consistency, test-retest reliability, intra-rater reliability, inter-rater reliability, or measurement error) and one type of validity (structural validity, hypotheses testing, cross-cultural validity, or criterion validity): the FPI [28], the SDM-Q-9 original German version [29], the SDM-Q-9 Spanish version [30–32] the SDM-Q-9 Dutch version,[33] the SDM-Q-9-PSY in Hebrew [34], the SDM-Q-doc original German version,[35] and the SDM-Q-doc Dutch version [33]. Of these instruments however, the SDM-Q-9 Spanish version [30–32], the SDM-Q-9-PSY in Hebrew[34] and the SDM-Q-doc original German version,[35] are the only instruments without any negative evidence on other measurement properties. In the online Supporting Information (S2 Table), we present the separate ratings for each included article, for both the appraisal of the methodological quality and the quality of measurement properties.

**Table 6:** Best level of evidence for each measurement property *per instrument* measuring the process of SDM (N = 38).

Instrument [reference(s) to validation study]	# evalua-tion studies	Internal consistency		Test retest reliability		Inter- rater reliability		Intra- rater reliability		Content validity		Structural validity/ Item response theory (IRT)		Hypotheses testing		Cross-cultural validity		Criterion validity		Responsive-ness	
		#	S	#	S	#	S	#	S	#	S	#	S	#	S	#	S	#	S	#	S
**Patient questionnaires (N = 12)**																					
PPC [66]	1	n.a.		0		n.a.		n.a.		0		n.a.		1	?	n.a.		0		0	
CPSpost [66, 67]	2	n.a.		0		n.a.		n.a.		0		n.a.		2	++	n.a.		0		0	
FPI [28]	1	1	++	1	+	n.a.		n.a.		0		1	++	1	-	n.a.		0		0	
COMRADE [38, 68]	2	1	?	0		n.a.		n.a.		0		2	--	2	-	n.a.		0		0	
SDM-Q [39]	1	n.a.		0		n.a.		n.a.		0		IRT:1	IRT:--	1	?	n.a.		0		0	
SDM-Q-9 [29, 69]	2	2	+++	0		n.a.		n.a.		0		1	+++	1	--	n.a.		0		0	
SDM-Q-9 (Spanish) [30–32]	3	3	+++	0		n.a.		n.a.		1	?	2 / IRT:1	+++ / IRT:++	0		2	+	0		0	
SDM-Q-9 (Dutch) [33]	1	1	+++	0		n.a.		n.a.		0		1	+++	1	-	0		0		0	
SDM-Q-9 Psy (Hebrew) [34]	1	1	++	0		n.a.		n.a.		0		1	++	1	+	0		0		0	
SDM-Q-9 (English) [30]	1	1	+++	0		n.a		n.a		0		0		0		1	?	0		0	
CollaboRATE [70]	1	n.a.	0		n.a.	1		++		0		n.a.	1	-	n.a.	0		1	?		
CollaboRATE (Swedish) [41]	1	1	?	1	-	n.a		n.a		0		0		1	-	0		0		0	
SMDMQ (Taiwanese) [42, 43]*	1	n.a.		0		n.a.		n.a.		1	?	IRT:1	IRT:--	0		n.a.		0		0	
SDM Process Score [44]	1	0		0		n.a.		n.a.		0		0		1	+	n.a.		0		0	
MADM [45]	1	1	+++	0		n.a		n.a		0		1	?	1	?	n.a		0		0	
Dyadic OPTION patient version (Swedish) [41]	1	1	?	1	-	n.a		n.a		0		0		1	-	0		0		0	
**Provider questionnaires (N = 3)**																					
SDM-Q-Doc [35]	1	1	+++	0		n.a.		n.a.		0		1	+++	0		n.a.		0		0	
SDM-Q-Doc (Persian) [46]	1	1	?	1	?	n.a.		n.a.		0		0		0		0		0		0	
SDM-Q-Doc (Dutch) [33]	1	1	+++	0		n.a.		n.a.		0		1	+++	1	-	0		0		0	
SDM-Q-Doc (Spanish) [47]	1	1	?	0		n.a.		n.a.		1	?	1	?	1	?	0		0		0	
**Observer-based coding schemes (N = 18)**																					
IDM [71]	1	0		n.a.		0		0		0		0		1	-	n.a.		0		0	
DSAT [49, 72]	2	0		n.a.		1	?	0		0		0		2	+/-	n.a.		0		0	
DSAT-10 [50]	1	0		n.a.		1	--	0		0		0		0		n.a.		0		0	
OPTION [51, 72]	2	0	?	n.a.		1	--	1	?	0		1	--	2	--	n.a.		0		0	
OPTION (revised)[52, 71, 73, 74]	4	1	?	n.a.		3	--	2	?	0		1	---	2	-	n.a.		0		0	
OPTION (Italian) [53]	1	1	+	n.a.		1	+	1	+	0		1	?	0		0		0		0	
OPTION (revised)(German) [54]	1	1	?	n.a.		1	-	0		0		1	++	1	-	0		0		0	
OPTION (revised and modified)(German) [55]	1	1	?	n.a.		1	?	0		0		0		1	?	0		0		0	
OPTION^12^ (Dutch) [56]	1	0		n.a.		1	?	0		0		0		0		0		0		0	
Observer OPTION^5 item^ [74, 75]	2	n.a.		n.a.		2	--	1	?	0		n.a.		2	++	n.a.		0		0	
OPTION^5^ (Dutch) [56]	1	n.a.		n.a.		1	?	0		0		n.a.		1	+	0		0		0	
RPAD [58]	1	0		n.a.		m	m	m	m	0		0		1	-	n.a.		0		0	
DAS-O [59, 72]**	1	0		n.a.		1	?	1	?	1	++	0		1	- ***	n.a.		0		0	
SDM Scale [60]	1	1	?	n.a.		1	?	1	?	0		1	?	1	+	n.a.		0		0	
PES [no validation study published]	0	0		n.a.		0		0		0		0		0		n.a.		0		0	
DEEP-SDM [no validation study published]	0	0		n.a.		0		0		0		0		0		n.a.		0		0	
Shared decision-making rating [63]	1	0		n.a.		1	?	0		0		0		0		n.a.		0		0	
Mappin'SDM_norge_ (Norwegian) [64]	1	0		n.a.		1	-	0		0		0		1	-	0		1	?	0	
**Mixed instruments (N = 2 measuring N = 5 different perspectives)**																					
dyadic OPTION^Patient^ [26]	1	0		0		n.a.		n.a.		0		0		1	-	n.a.		0		0	
dyadic OPTION^Clinician^[26]	1	0		0		n.a.		n.a.		0		0		1	+	n.a.		0		0	
MAPPIN'SDM patient questionnaire [27, 76] ****	1	1	?	0		n.a.		n.a.		1	++	0		1	-	n.a.		0		0	
MAPPIN'SDM doctor questionnaire [27, 76] ****	1	1	?	0		n.a.		n.a.		1	++	0		1	-	n.a.		0		0	
MAPPIN'SDM coding scheme [27, 76, 77] ****	2	0		n.a.		2	--	0		0		0		2	--	n.a.		0		0	

S = result of best-evidence synthesis. n.a. = the measurement property is not applicable to this instrument. m = missing. Rating: +++/---Strong level of evidence for positive/negative results, ++/--Moderate level of evidence for positive/negative results, +/- Limited evidence for positive/negative results, +/- Conflicting evidence,? = Unknown, due to poor methodological quality, empty cell = No synthesis possible due to a lack of validation studies for this measurement property. Measurement error was left out from the Table because it has not been evaluated for any of the instruments included.

# = number of studies on which the best-evidence synthesis regarding the measurement property was based

*References [42] and reference [43] and both presents results of the development and validation for the SMDMQ (Taiwanese), however the results presented seem the exact same in both articles, reference [43] was therefore left out in the data extraction and analysis and also not included in the number of included articles.

** Reference [59] and reference [72] both present hypotheses testing for the DAS-0, however reference [72] was based on the same dataset as reference [59], therefore reference [72] was left out for the evidence synthesis of the DAS-O.

*** There is a negative score for hypotheses testing because the authors had hypothesized that correlations would be medium-sized but they actually found strong relationships with instruments measuring the same construct.

**** Reference [27] and Reference [76] and both present results about internal consistency for the MAPPIN'SDM patient and doctor questionnaire and about inter-rater reliability for the MAPPIN'SDM coding scheme, however reference [76] made use of the same dataset as reference [27]; results of reference [76]were therefore left out for the evidence synthesis of the Mappin’SDM.

### 3.3 Overall results for the quality of validation studies and measurement properties

In the next three sections we will describe overall results on the quality of included studies and instruments, beginning with an overview of measurement properties that have been evaluated for the included instruments (section 3.3.1), the overall results on the methodological quality of the included validation studies (section 3.3.2), and overall results on the best-evidence synthesis (section 3.3.3). To allow for generalization, we present overall results only for measurement properties that have been evaluated in at least five studies (section 3.3.2) or for at least five instruments (section 3.3.3). We do not present overall results on the quality rating of measurement properties (see Methods section 2.4.2), because we regard them as being irrelevant without the correction for methodological quality. The results on the measurement properties evaluation for each included article and each instrument evaluated in the articles can be found in the online Supporting Information (S1 Table)

#### 3.3.1 Overall results on which measurement properties are evaluated

The measurement property evaluation results are presented in Table 7. The number of instruments for which each of the different measurement properties have been evaluated, taking into account whether the property was applicable or not, is presented in Table 7, column 2 and 3. Two measurement properties were evaluated in more than two-thirds of the instruments: hypotheses testing, and intra-rater reliability in case of coding schemes. Seven measurement properties were evaluated for in less than one-third of instruments: Test-retest reliability, measurement error, content validity, cross-cultural validity, criterion validity, responsiveness, and the *floor and ceiling effects* and *minimal important change values*, *both* aspects of interpretability. Of note, internal consistency and structural validity were evaluated for a majority of questionnaires, but a minority of coding schemes.

**Table 7:** Overall results on best-evidence synthesis *per measurement property* of instruments measuring the process of SDM (N = 43).

Measurement property	Applicable to instruments	Evaluated for instruments		Overall level of evidence							
				Unknown		Negative*		Conflicting		Positive*	
	N	N	(%)	N	(%)	N	(%)	N	(%)	N	(%)
**Internal consistency**											
Total	36	22	(61)	**12**	**(55)**	0	(0)	0	(0)	10	(46)
Questionnaires	19	16	(84)	**7**	(44)	0	(0)	0	(0)	**9**	**(56)**
Coding schemes	17	6	(35)	**5**	**(83)**	0	(0)	0	(0)	1	(17)
**Test retest reliability**											
Total	24	4	(17)	-	-	-	-	-	-	-	-
Questionnaires	24	4	(17)	-	-	-	-	-	-	-	-
Coding schemes	0	n.a.	n.a.	-	-	-	-	-	-	-	-
**Inter-rater reliability**											
Total	19	15	(79)	**7**	(47)	7	(47)	0	(0)	1	(7)
Questionnaires	0	n.a.	n.a.	-	-	-	-	-	-	-	-
Coding schemes	19	15	(79)	**7**	(47)	7	(47)	0	(0)	1	(7)
**Intra-rater reliability**											
Total	19**	7	(37)	**5**	**(71)**	0	(0)	0	(0)	2	(29)
Questionnaires	0**	1	n.a.	-	-	-	-	-	-	-	-
Coding schemes	19	6	(33)	**5**	**(83)**	0	(0)	0	(0)	1	(17)
**Measurement error**											
Total	43	0	(0)	-	-	-	-	-	-	-	-
Questionnaires	43	0	(0)	-	-	-	-	-	-	-	-
Coding schemes	43	0	(0)	-	-	-	-	-	-	-	-
**Content validity**											
Total	43	6	(14)	**3**	**(50)**	0	(0)	0	(0)	**3**	**(50)**
Questionnaires	24	5	(21)	**3**	**(50)**	0	(0)	0	(0)	**3**	**(50)**
Coding schemes	19	1	(5)	-	-	-	-	-	-	-	
**Structural validity**											
Total	36	15	(42)	4	(27)	3	(20)	0	(0)	**8**	**(53)**
Questionnaires	19	10	(56)	2	(20)	1	(10)	0	(0)	**7**	**(70)**
Coding schemes	17	5	(29)	2	(40)	20	(40)	0	(0)	1	(20)
**Hypotheses testing**											
Total	43	32	(74)	5	(16)	**19**	**(59)**	1	(43)	7	(22)
Questionnaires	24	19	(79)	4	(21)	**11**	**(58)**	0	(0)	4	(21)
Coding schemes	19	13	(68)	1	(8)	**8**	**(62)**	1	(8)	3	(23)
**Cross-cultural validity**											
Total	15	2	(13)	-	-	-	-	-	-	-	-
Questionnaires	9	2	(22)	-	-	-	-	-	-	-	-
Coding schemes	6	0	(0)	-	-	-	-	-	-	-	-
**Criterion validity**											
Total	43	1	(2)	-	-	-	-	-	-	-	-
Questionnaires	24	0	(0)	-	-	-	-	-	-	-	-
Coding schemes	19	1	(5)	-	-	-	-	-	-	-	-
**Responsiveness**											
Total	43	1	(2)	-	-	-	-	-	-	-	-
Questionnaires	24	1	(4)	-	-	-	-	-	-	-	-
Coding schemes	19	0	(0)	-	-	-	-	-	-	-	-
**Interpretability: Floor and ceiling effects**											
Total	43	11	(26)	-	-	-	-	-	-	-	-
Questionnaires	24	7	(29)	-	-	-	-	-	-	-	-
Coding schemes	19	4	(21)	-	-	-	-	-	-	-	-
**Interpretability: Minimal important change**											
Total	43	0	(0)	-	-	-	-	-	-	-	-
Questionnaire	24	0	(0)	-	-	-	-	-	-	-	-
Coding schemes	19	0	(0)	-	-	-	-	-	-	-	-

Color-coding is used to indicate that a specific measurement property had a particular direction of the best level of in ≥ 50% of instruments evidence (blue = unknown, red = negative and green = positive) and the best evidence synthesis was performed for at least five instruments. n.a. = not applicable

* Results in negative or positive direction have either a “limited”, “moderate” or “strong” level of evidence, based on the best-evidence synthesis.

** The measurement property intra-rater reliability is usually not applicable to questionnaires. Authors of one questionnaire have used this type of evaluation as an alternative for test-retest reliability assessment.

#### 3.3.2 Overall results on the methodological quality of included validation studies

The methodological quality used was excellent or good in at least half of the studies for the measurement properties of content validity (50%) and structural validity (82%) (Table 8). The methodological quality was poor in at least half of the studies for the measurement properties of internal consistency (52%), inter-rater reliability (53%), intra-rater reliability (75%), and content validity (50%). The quality of validation studies was more often good or excellent for questionnaires than for coding schemes with regard to internal consistency (58% in case of questionnaires, none in case of coding schemes) and structural validity (92% in case of questionnaires, 40% in case of coding schemes). A rating of “poor” in the quality assessment of internal consistency testing was most often due to a lack of factor analysis (COSMIN checklist for internal consistency, item 5) or lack of an internal consistency statistic for subscales (COSMIN checklist for internal consistency, item 7). For inter- and intra-rater reliability testing, a rating of “poor” was most often due to small sample sizes (COSMIN checklist for reliability, item 3) or to the application of statistical methods that were inappropriate for the measurement level of the scale (COSMIN checklist for reliability, items 11–14).

**Table 8:** Overall results on methodological quality *of the studies* that evaluated measurement properties of instruments measuring the process of SDM, as based on COSMIN checklist scoring.

Measurement property	Total number of assessments	Methodological quality rating							
		Poor		Fair		Good		Excellent	
		N	(%)	N	(%)	N	(%)	N	(%)
**Internal consistency**									
Total	25	**13**	**(52)**	1	(4)	5	(20)	6	(24)
Questionnaires	19	**8**	**(42)**	0	-	5	(26)	6	(32)
Coding schemes	6	**5**	**(83)**	1	(17)	0	-	0	-
**Inter-rater reliability**									
Total	19	**10**	**(53)**	4	(21)	5	(26)	0	-
Questionnaires	0								
Coding schemes	19	**10**	**(53)**	4	(21)	5	(26)	0	-
**Intra-rater reliability**									
Total	8	**6**	**(75)**	1	(13)	1	(13)	0	-
Questionnaires	1	0	-	0	-	1	(100)	0	-
Coding schemes	7	**6**	**(86)**	1	(14)	0	-	0	-
**Content validity**									
Total	6	**3**	**(50)**	0	-	**3**	**(50)**	0	-
Questionnaires	5	3	(60)	0	-	2	(40)	0	-
Coding schemes	1	0	-	0	-	1	(100)	0	-
**Structural validity**									
Total	17	2	(12)	1	(6)	**8**	**(47)**	**6**	**(35)**
Questionnaires	12	0	-	1	(8)	**6**	**(50)**	**5**	**(42)**
Coding schemes	5	2	(40)	0	-	1	(20)	1	(20)
**Hypotheses testing**									
Total	39	8	(21)	**26**	**(67)**	45	(13)	0	-
Questionnaires	21	5	(24)	**13**	**(62)**	3	(14)	0	-
Coding schemes	18	3	(17)	**13**	**(72)**	2	(11)	0	-

Color-coding is used to indicate that the assessment of a specific measurement property had a particular level of quality in ≥ 50% of studies (red = poor, yellow = fair and green = good or excellent) and the assessment had been done in at least five studies; we summed the categories ‘good’ and ‘excellent’ for this purpose.

#### 3.3.3 Overall results on the best evidence synthesis of included *instruments*

The best available evidence was unknown for 50% or more of the instruments for the measurement properties of internal consistency, intra-rater reliability, and content validity due to poor methods (Table 7). For two measurement properties, the best available evidence indicated positive results (limited, moderate, or strong) for 50% or more of the instruments: Content validity and structural validity. The best available evidence indicated negative results (limited, moderate, or strong) for hypotheses testing for 59% of the instruments and for intra-rater reliability for 47% of the instruments. Results for questionnaires were overall more positive and for coding-schemes more often unknown regarding internal consistency and structural validity.

## 4. Discussion

The aim of this systematic review was to provide an overview of the measurement quality of existing instruments measuring the process of SDM. In total, 40 instruments were included in our analysis; primarily patient questionnaires or observer-based coding schemes, but also a few provider questionnaires and ‘mixed’ instruments. There is a general lack of evidence for the appraisal of most measurement properties. This is either because the property was not evaluated, or because the methodology applied was of poor quality. The best-evidence synthesis indicated positive results for at least half of the instruments that have investigated content validity (50%) and structural validity (53%), but negative results for a major part of instruments that have been evaluated for inter-rater reliability (47%) and hypotheses testing (59%). We will highlight the results that in our opinion are most relevant for further validation of existing instruments and the development of new instruments, and provide recommendations for future research.

### 4.1 Lack of detailed description and assessment of the construct

During data extraction, we noticed that instrument developers often only provided a vague definition of the construct being measured or none at all. Furthermore, or as a consequence of this, for only 14% of the instruments content validity testing was described, (including assessment of item relevance and comprehensiveness of the item set for the measured construct). Additionally, the underlying measurement model was made explicit for only two instruments, with a formative model applied in both instances. The major difference between reflective and formative models is the direction of causality between the construct and its items. In formative models the latent construct of interest is a result of independent items measured (causal indicators), whereas in reflective models the latent construct determines the items (effect indicators) being measured [78, 79]. Therefore, exploratory factor analysis and internal consistency are only relevant for reflective models. In 2011, Wollschläger called upon the SDM field to reach consensus on the most suitable underlying model [80], but it appears that the field is only slowly responding to this call. For most questionnaires, the authors apparently assumed a reflective model, as they assessed factor structure and/or internal consistency. However, this practice may have resulted from a lack of a clear definition of the construct, which is needed to correctly specify the underlying measurement model (see Jarvis et al 2003, Table 3) [78], or from the assumption that assessing these properties is required, even when inappropriate. Following the steps Jarvis presents to decide on the most suitable model, we suggest that it may be more suitable to assume a formative model to measure the process of SDM. Definitions of the SDM process often contain required but independent steps, each of which do not necessarily relate to each other. Changes in one or more of these steps result in changes in levels of SDM, but changes in SDM are not necessarily reflected in changes in all items. That is, a physician explaining that a decision has to be made will increase measures of the SMD process, but increases in the SDM process will not necessarily be reflected in a physician explaining that a decision has to be made. Choosing a formative model has implications for the development of an instrument, as factor structure and internal consistency are not relevant to determine validity of instruments with formative models, and thus cannot inform the selection of the items. For instrument with formative models, content validity testing is therefore even more relevant to make the final selection of items. We want to stress the importance of a clear construct definition and sound content validity testing as a first step in the development and validation of measurement instruments. In any case, the choice of the underlying model should be explicitly described.

### 4.2 Lack of stability

Test-retest evaluations of questionnaires were performed infrequently (for 17% of questionnaires). The main barrier might be that it cannot be assumed that patients’ and providers’ views are stable between test and retest. Decisions might have been made and/or acted upon which can bias how participants look back on decision processes. Despite these barriers, from a psychometric point of view, lack of stability evaluations of the questionnaires compromises the interpretation of questionnaire results. As an alternative, the developers of the CollaboRATE used analogue patients to determine the intra-rater reliability of their questionnaire [70]. Investigating the validity of this and other methods as possible equivalents for test-retest reliability testing may prove valuable for psychometric testing of SDM measures.

Inter-rater reliability of coding scheme scores has often been assessed but these assessments frequently show negative results, raising questions about the stability of the scores. Caution should be applied when comparing observer scores between studies when intra-rater reliability is poor. Training might improve agreement between the coders within a study. However, training does not automatically improve inter-rater agreement between research groups. More detailed definitions of items and response scales and more frequent consensus discussions throughout the coding process limit the opportunity for subjective interpretation of the items, and thus might improve inter-rater reliability further.

### 4.3 Hypotheses testing: Poor results or poor hypotheses?

The best-evidence synthesis showed that results on hypotheses testing, as a means to assess construct validity, indicated negative results for more than half of the instruments for which this had was evaluated. The hypotheses tested (see online supporting information S1 Table) that were not confirmed often assessed relationships with instruments that measure (slightly) different constructs (e.g., satisfaction with decision, patients’ information seeking preference, anxiety). Also, hypotheses about relationships with instruments that measure the same construct, whether measured from the same or from a different perspective, were often not confirmed or did not reach the threshold for positive results for correlation coefficients of ≥0.50. This leads us to conclude that poor results for hypotheses testing might reveal methodological problems regarding the suitability of comparators that authors have chosen–which is not accounted for in our COSMIN rating. Until we reach consensus on how to define the process of SDM and on whether SDM viewed from the perspective of the provider, patient, or observer can be regarded as the same construct, authors should be careful in formulating hypotheses for construct validity testing. A good alternative for hypotheses testing about the relationship between instruments that define the construct differently or that measure the same construct but from another perspective could be to assess known group differences.

### 4.4 Lack of insight into the ability to measure change and to interpret change

Measurement properties relevant to the validity and interpretation of change scores have barely been studied. This is in line with what Scholl et al. already concluded in 2011. Measurement error, responsiveness (evaluated once but using poor methods [70]) and minimal important change values are unknown for the instruments included, even though they are indispensable for interpreting results of intervention studies. Anchor-based methods that make use of an external criterion [81] are well-suited to determine which change is regarded as relevant in terms of important improvements or deteriorations of the process of SDM. Another obstacle however is that the determination of measurement error is essential for the interpretation of minimal important change values, but its determination might face the same barriers as the test-retest evaluation.

### 4.5 Strengths and limitations of the review

A first strength of our study was the comprehensive search in multiple online databases, for which we set no time limits on publication date, nor did we exclude any type of instrument (i.e. *patient* questionnaires, *provider* questionnaires or *observer* based coding schemes). Second, two raters and when necessary three, evaluated the eligibility of articles, extracted the data, and performed the quality appraisal for each measurement property. We therefore expect our results to be highly valid. Third, to provide an unbiased appraisal of the measurement quality of included instruments, we took into account the results and methodological quality of all their validation studies for the best-evidence synthesis and we rated methodological quality based on the widely-accepted COSMIN standards. Fourth, due to the high number of included instruments, we were able to provide insight into overall trends on the existence of measurement property evaluations, their quality, and the overall quality of instruments. This insight makes it possible to provide general recommendations on how to improve the quality of SDM process instruments and their validation studies.

Our study has some limitations. First, to be eligible for inclusion an article must describe a study that aimed to develop a SDM-process instrument or that validates a SDM-process instrument. We might have missed relevant articles if development or validation of an instrument was not explicitly mentioned in either its title or its abstract. Second, an overrepresentation of data may have biased our best-evidence synthesis. That is, the number of validation studies influences the rating of the best level of evidence and strictly speaking, one should correct this number for those instances when validation studies have been performed once, but authors have published about the same data in multiple articles, but with slightly different foci. After contacting authors, we corrected for this phenomenon twice, i.e., for the DAS-O and the Mappin’SDM (see the footnotes underneath Table 6). However, we cannot state with certainty that overrepresentation is not at stake for other instruments. We recommend more explicit reporting of multiple data use when publishing secondary analyses. Third, our analysis was limited to the evaluation of the measurement properties of existing SDM process instruments. It does not include a detailed analysis of the content of these instruments. To gain more insight into what exactly they measure and what not, further research on the operationalization of existing SDM process instruments is needed. Furthermore, our quality evaluation of SDM process instruments is only applicable for research settings and at a group level. No conclusions can be drawn on the suitability of these instruments for other purposes, such as for the evaluation of individual healthcare providers’ SDM skills. With the current emphasis on value-based healthcare, the applicability of instruments measuring the process of SDM within routine clinical settings needs to be investigated in future research.

### 4.6 Conclusions

A large number of instruments are available to assess the SDM process, but, evidence is lacking regarding the measurement quality of these instruments, partly because measurement properties have not been evaluated at all, partly because the validation studies are of poor quality. Clearly, this does not imply that existing instruments measuring the process of SDM are of poor quality, but that often their quality is unknown. In practice, the choice for the most appropriate instrument can therefore best be based on the content of the instrument and other characteristics of the instruments that suit best the aim of the study and the resources available for the study, such as the perspective that is assessed and the number of items. We suggest the following recommendations for quality improvement of existing instruments and their validation studies:

- Provide a clear definition of the construct of SDM process.
- Perform content validity analyses prior to further validation.
- Include large-enough sample sizes in validation studies; improvement of sample sizes is especially needed for inter- and intra-rater reliability testing of coding schemes.
- Seek alternative ways to evaluate test-retest reliability of questionnaires for the process of SDM.
- Find ways to improve inter-rater reliability of coding schemes; e.g., by providing more detailed descriptions of coding scheme items.
- Include constructs that are as similar as possible to the process of SDM when formulating hypotheses to evaluate construct validity, and, alternatively, make use of known-group differences testing.
- Determine minimal important change values to inform the interpretation of change scores in intervention studies.

Above all, we recommend to further evaluate and refine existing instruments and to adhere as best as possible to the COSMIN guidelines [20, 21, 23] to help guarantee high-quality evaluations.

## Appendix A: Search strategy

### PubMed

((("Decision Making"[majr:noexp] OR decision making[tiab] OR decision making[ot] OR decisionmaking[tiab] OR decisionmaking[ot]) AND (professional-patient relations[majr] OR ((Patient[tiab]) AND (provider[tiab] OR physician[tiab] OR professional[tiab] OR doctor[tiab]) AND (relation[tiab] OR relations[tiab] OR contact[tiab] OR communication[tiab] OR interaction[tiab] OR interactions[tiab])) OR ((Patient[ot]) AND (provider[ot] OR physician[ot] OR professional[ot] or doctor[ot]) AND (relation[ot] OR relations[ot] OR contact[ot] OR communication[ot] OR interaction[ot] OR interactions[ot])) OR Patient participation[majr] OR Patient Participation[tiab] OR patient participation[ot] OR patients participation[tiab] OR patients participation[ot] OR patient's participation[tiab] OR patient's participation[ot] OR patient involvement[tiab] OR patient involvement[ot] OR patients involvement[tiab] OR patients involvement[ot] OR patient's involvement[tiab] OR patient's involvement[ot] OR consultation*[tiab] OR encounter[tiab] OR consultation*[ot] OR encounter[ot])) OR shared decision[tiab] OR shared decision[ot] OR shared decisions[tiab] OR shared decisions[ot] OR shared decisionmaking[tiab] OR shared decisionmaking[ot] OR SDM[tiab] OR SDM[ot] OR Shared medical decision[tiab] OR Shared medical decision[ot] OR Shared treatment decision[tiab] OR Shared treatment decision[ot] OR Shared medical decisions[tiab] OR Shared medical decisions[ot] OR Shared treatment decisions[tiab] OR Shared treatment decisions[ot] OR Shared clinical decision[tiab] OR Shared clinical decision[ot] OR Shared clinical decisions[tiab] OR Shared clinical decisions[ot])

AND

(Health Care Surveys [majr:noexp] OR "Outcome and Process Assessment (Health Care)"[majr:noexp] OR "Outcome Assessment(Health Care)"[majr:noexp] OR "Patient Outcome Assessment"[majr:noexp] OR "Questionnaires"[majr] OR scale[tiab] OR scale[ot] OR scales[tiab] OR scales[ot] OR instrument[tiab] OR instrument[ot] OR instruments[tiab] OR instruments[ot] OR questionnaire[tiab] OR questionnaire[ot] OR questionnaires[tiab] OR questionnaires[ot] OR survey[tiab] OR survey[ot] OR surveys[tiab] OR surveys[ot] OR assess*[tiab] OR assess*[ot] OR coding scheme[tiab] OR coding scheme[ot] OR coding schemes[tiab] OR codingscheme[tiab] OR codingscheme[ot] OR codingschemes[tiab] OR codingschemes[ot] OR rating[tiab] OR rating[ot] OR ratings[tiab] OR ratings[ot] OR selfreport[tiab] OR selfreport[ot] OR self report[tiab] OR self report[ot] OR selfreports[tiab] OR selfreports[ot] OR self reports[tiab] OR self reports[ot] OR "Checklist"[majr] OR measure[tiab] OR measure[ot] OR measures[tiab] OR measures[ot] OR "observation"[majr] OR observation[tiab] OR observation[ot] OR observations[tiab] OR observations[ot])

AND

(instrumentation[sh] OR methods[sh] OR Validation Studies[pt] OR Comparative Study[pt] OR "psychometrics"[82] OR psychometr*[tw] OR clinimetr*[tw] OR clinometr*[tw] OR "outcome assessment (health care)"[82] OR outcome assessment[tw] OR outcome measure*[tw] OR "observer variation"[82] OR observer variation[tiab] OR "Health Status Indicators"[82] OR "reproducibility of results"[82] OR reproducib*[tiab] OR "discriminant analysis"[82] OR reliab*[tiab] OR unreliab*[tiab] OR valid*[tiab] OR coefficient[tiab] OR homogeneity[tiab] OR homogeneous[tiab] OR "internal consistency"[tiab] OR (cronbach*[tiab] AND (alpha[tiab] OR alphas[tiab])) OR (item[tiab] AND (correlation*[tiab] OR selection*[tiab] OR reduction*[tiab])) OR agreement[tiab] OR precision[tiab] OR imprecision[tiab] OR "precise values"[tiab] OR test–retest[tiab] OR (test[tiab] AND retest[tiab]) OR (reliab*[tiab] AND (test[tiab] OR retest[tiab])) OR stability[tiab] OR interrater[tiab] OR inter-rater[tiab] OR intrarater[tiab] OR intra-rater[tiab] OR intertester[tiab] OR inter-tester[tiab] OR intratester[tiab] OR intra-tester[tiab] OR interobserver[tiab] OR inter-observer[tiab] OR intraobserver[tiab] OR intra-observer[tiab] OR intertechnician[tiab] OR inter-technician[tiab] OR intratechnician[tiab] OR intra-technician[tiab] OR interexaminer[tiab] OR inter-examiner[tiab] OR intraexaminer[tiab] OR intra-examiner[tiab] OR interassay[tiab] OR inter-assay[tiab] OR intraassay[tiab] OR intra-assay[tiab] OR interindividual[tiab] OR inter-individual[tiab] OR intraindividual[tiab] OR intra-individual[tiab] OR interparticipant[tiab] OR inter-participant[tiab] OR intraparticipant[tiab] OR intra-participant[tiab] OR kappa[tiab] OR kappa’s[tiab] OR kappas[tiab] OR repeatab*[tiab] OR ((replicab*[tiab] OR repeated[tiab]) AND (measure[tiab] OR measures[tiab] OR findings[tiab] OR result[tiab] OR results[tiab] OR test[tiab] OR tests[tiab])) OR generaliza*[tiab] OR generalisa*[tiab] OR concordance[tiab] OR (intraclass[tiab] AND correlation*[tiab]) OR discriminative[tiab] OR "known group"[tiab] OR factor analysis[tiab] OR factor analyses[tiab] OR dimension*[tiab] OR subscale*[tiab] OR (multitrait[tiab] AND scaling[tiab] AND (analysis[tiab] OR analyses[tiab])) OR item discriminant[tiab] OR interscale correlation*[tiab] OR error[tiab] OR errors[tiab] OR "individual variability"[tiab] OR (variability[tiab] AND (analysis[tiab] OR values[tiab])) OR (uncertainty[tiab] AND (measurement[tiab] OR measuring[tiab])) OR "standard error of measurement"[tiab] OR sensitiv*[tiab] OR responsive*[tiab] OR ((minimal[tiab] OR minimally[tiab] OR clinical[tiab] OR clinically[tiab]) AND (important[tiab] OR significant[tiab] OR detectable[tiab]) AND (change[tiab] OR difference[tiab])) OR (small*[tiab] AND (real[tiab] OR detectable[tiab]) AND (change[tiab] OR difference[tiab])) OR meaningful change[tiab] OR "ceiling effect"[tiab] OR "floor effect"[tiab] OR "Item response model"[tiab] OR IRT[tiab] OR Rasch[tiab] OR "Differential item functioning"[tiab] OR DIF[tiab] OR "computer adaptive testing"[tiab] OR "item bank"[tiab] OR "cross-cultural equivalence"[tiab])

NOT

("addresses"[Publication Type] OR "biography"[Publication Type] OR "case reports"[Publication Type] OR "comment"[Publication Type] OR "directory"[Publication Type] OR "editorial"[Publication Type] OR "festschrift"[Publication Type] OR "interview"[Publication Type] OR "lectures"[Publication Type] OR "legal cases"[Publication Type] OR "legislation"[Publication Type] OR "letter"[Publication Type] OR "news"[Publication Type] OR "newspaper article"[Publication Type] OR "patient education handout"[Publication Type] OR "popular works"[Publication Type] OR "congresses"[Publication Type] OR "consensus development conference"[Publication Type] OR "consensus development conference, nih"[Publication Type] OR "practice guideline"[Publication Type]) NOT ("animals"[MeSH Terms] NOT "humans"[MeSH Terms])

### Embase

(((*"Decision Making"/ OR decision making.ti,ab OR decisionmaking.ti,ab) AND ("doctor patient relation"/ OR "nurse patient relationship"/ OR ((Patient.ti,ab) AND (provider.ti,ab OR physician.ti,ab OR professional.ti,ab OR doctor.ti,ab) AND (relation.ti,ab OR relations.ti,ab OR contact.ti,ab OR communication.ti,ab OR interaction.ti,ab OR interactions.ti,ab)) OR ((Patient.mp) AND (provider.mp OR physician.mp OR professional.mp OR doctor.mp) AND (relation.mp OR relations.mp OR contact.mp OR communication.mp OR interaction.mp OR interactions.mp)) OR *"Patient participation"/ OR Patient Participation.ti,ab OR patients participation.ti,ab OR patient's participation.ti,ab OR patient involvement.ti,ab OR patients involvement.ti,ab OR patient's involvement.ti,ab OR consultation*.ti,ab OR encounter.ti,ab)) OR shared decision.ti,ab OR shared decisions.ti,ab OR shared decisionmaking.ti,ab OR SDM.ti,ab OR Shared medical decision.ti,ab OR Shared treatment decision.ti,ab OR Shared medical decisions.ti,ab OR Shared treatment decisions.ti,ab OR Shared clinical decision.ti,ab OR Shared clinical decisions.ti,ab)

AND

(*"Health Care Survey"/ OR *"Outcome Assessment"/ OR exp *"Questionnaire"/ OR scale.ti,ab OR scale.mp OR scales.ti,ab OR scales.mp OR instrument.ti,ab OR instrument.mp OR instruments.ti,ab OR instruments.mp OR questionnaire.ti,ab OR questionnaire.mp OR questionnaires.ti,ab OR questionnaires.mp OR survey.ti,ab OR survey.mp OR surveys.ti,ab OR surveys.mp OR assess*.ti,ab OR assess*.mp OR coding scheme.ti,ab OR coding scheme.mp OR coding schemes.ti,ab OR codingscheme.ti,ab OR codingscheme.mp OR codingschemes.ti,ab OR codingschemes.mp OR rating.ti,ab OR rating.mp OR ratings.ti,ab OR ratings.mp OR selfreport.ti,ab OR selfreport.mp OR self report.ti,ab OR self report.mp OR selfreports.ti,ab OR selfreports.mp OR self reports.ti,ab OR self reports.mp OR "self report"/ OR *"Checklist"/ OR measure.ti,ab OR measure.mp OR measures.ti,ab OR measures.mp OR *"Observation"/ OR observation.ti,ab OR observation.mp OR observations.ti,ab OR observations.mp)

AND

(exp "intermethod comparison"/ OR exp "data collection method"/ OR exp "validation study"/ OR exp "feasibility study"/ OR exp "pilot study"/ OR exp "psychometry"/ OR exp "reproducibility"/ OR reproducib*.ti,ab OR "audit".ti,ab OR psychometr*.mp OR clinimetr*.ti,ab OR clinometr*.ti,ab OR exp "observer variation"/ OR "observer variation".ti,ab OR exp "discriminant analysis"/ OR exp "validity"/ OR reliab*.ti,ab OR valid*.ti,ab OR "coefficient".ti,ab OR "internal consistency".ti,ab OR (cronbach*.ti,ab AND ("alpha".ti,ab OR "alphas".ti,ab)) OR "item correlation".ti,ab OR "item correlations".ti,ab OR "item selection".ti,ab OR "item selections".ti,ab OR "item reduction".ti,ab OR "item reductions".ti,ab OR "agreement".ti,ab OR "precision".ti,ab OR "imprecision".ti,ab OR "precise values".ti,ab OR "test-retest".ti,ab OR ("test".ti,ab AND "retest".ti,ab) OR (reliab*.ti,ab AND ("test".ti,ab OR "retest".ti,ab)) OR "stability".ti,ab OR "interrater".ti,ab OR "inter-rater".ti,ab OR "intrarater".ti,ab OR "intra-rater".ti,ab OR "intertester".ti,ab OR "inter-tester".ti,ab OR "intratester".ti,ab OR "intra-tester".ti,ab OR "interobeserver".ti,ab OR "inter-observer".ti,ab OR "intraobserver".ti,ab OR "intra-observer".ti,ab OR "intertechnician".ti,ab OR "inter-technician".ti,ab OR "intratechnician".ti,ab OR "intra-technician".ti,ab OR "interexaminer".ti,ab OR "inter-examiner".ti,ab OR "intraexaminer".ti,ab OR "intra-examiner".ti,ab OR "interassay".ti,ab OR "inter-assay".ti,ab OR "intraassay".ti,ab OR "intra-assay".ti,ab OR "interindividual".ti,ab OR "inter-individual".ti,ab OR "intraindividual".ti,ab OR "intra-individual".ti,ab OR "interparticipant".ti,ab OR "inter-participant".ti,ab OR "intraparticipant".ti,ab OR "intra-participant".ti,ab OR "kappa".ti,ab OR "kappas".ti,ab OR "coefficient of variation".ti,ab OR repeatab*.ti,ab OR (replicab*.ti,ab OR "repeated".ti,ab AND ("measure".ti,ab OR "measures".ti,ab OR "findings".ti,ab OR "result".ti,ab OR "results".ti,ab OR "test".ti,ab OR "tests".ti,ab)) OR generaliza*.ti,ab OR generalisa*.ti,ab OR "concordance".ti,ab OR ("intraclass".ti,ab AND correlation*.ti,ab) OR "discriminative".ti,ab OR "known group".ti,ab OR "factor analysis".ti,ab OR "factor analyses".ti,ab OR "factor structure".ti,ab OR "factor structures".ti,ab OR "dimensionality".ti,ab OR subscale*.ti,ab OR "multitrait scaling analysis".ti,ab OR "multitrait scaling analyses".ti,ab OR "item discriminant".ti,ab OR "interscale correlation".ti,ab OR "interscale correlations".ti,ab OR ("error".ti,ab OR "errors".ti,ab AND (measure*.ti,ab OR correlat*.ti,ab OR evaluat*.ti,ab OR "accuracy".ti,ab OR "accurate".ti,ab OR "precision".ti,ab OR "mean".ti,ab)) OR "individual variability".ti,ab OR "interval variability".ti,ab OR "rate variability".ti,ab OR "variability analysis".ti,ab OR ("uncertainty".ti,ab AND ("measurement".ti,ab OR "measuring".ti,ab)) OR "standard error of measurement".ti,ab OR sensitiv*.ti,ab OR responsive*.ti,ab OR ("limit".ti,ab AND "detection".ti,ab) OR "minimal detectable concentration".ti,ab OR interpretab*.ti,ab OR (small*.ti,ab AND ("real".ti,ab OR "detectable".ti,ab) AND ("change".ti,ab OR "difference".ti,ab)) OR "meaningful change".ti,ab OR "minimal important change".ti,ab OR "minimal important difference".ti,ab OR "minimally important change".ti,ab OR "minimally important difference".ti,ab OR "minimal detectable change".ti,ab OR "minimal detectable difference".ti,ab OR "minimally detectable change".ti,ab OR "minimally detectable difference".ti,ab OR "minimal real change".ti,ab OR "minimal real difference".ti,ab OR "minimally real change".ti,ab OR "minimally real difference".ti,ab OR "ceiling effect".ti,ab OR "floor effect".ti,ab OR "item response model".ti,ab OR "irt".ti,ab OR "rasch".ti,ab OR "differential item functioning".ti,ab OR "dif".ti,ab OR "computer adaptive testing".ti,ab OR "item bank".ti,ab OR "cross-cultural equivalence".ti,ab OR exp Comparative Study/ OR "Outcome assessment"/ OR outcome assessment.mp OR outcome measure*.mp OR exp "Health Status Indicators"/ OR homogeneity.ti,ab OR homogeneous.ti,ab)

NOT

("editorial"/ OR "letter"/ OR conference abstract.pt OR conference review.pt) NOT (exp "Animals"/ NOT exp "humans"/)

### Emcare

(((*"Decision Making"/ OR decision making.ti,ab OR decisionmaking.ti,ab) AND ("doctor patient relation"/ OR "nurse patient relationship"/ OR ((Patient.ti,ab) AND (provider.ti,ab OR physician.ti,ab OR professional.ti,ab OR doctor.ti,ab) AND (relation.ti,ab OR relations.ti,ab OR contact.ti,ab OR communication.ti,ab OR interaction.ti,ab OR interactions.ti,ab)) OR ((Patient.mp) AND (provider.mp OR physician.mp OR professional.mp OR doctor.mp) AND (relation.mp OR relations.mp OR contact.mp OR communication.mp OR interaction.mp OR interactions.mp)) OR *"Patient participation"/ OR Patient Participation.ti,ab OR patients participation.ti,ab OR patient's participation.ti,ab OR patient involvement.ti,ab OR patients involvement.ti,ab OR patient's involvement.ti,ab OR consultation*.ti,ab OR encounter.ti,ab)) OR shared decision.ti,ab OR shared decisions.ti,ab OR shared decisionmaking.ti,ab OR SDM.ti,ab OR Shared medical decision.ti,ab OR Shared treatment decision.ti,ab OR Shared medical decisions.ti,ab OR Shared treatment decisions.ti,ab OR Shared clinical decision.ti,ab OR Shared clinical decisions.ti,ab)

AND

(*"Health Care Survey"/ OR *"Outcome Assessment"/ OR exp *"Questionnaire"/ OR scale.ti,ab OR scale.mp OR scales.ti,ab OR scales.mp OR instrument.ti,ab OR instrument.mp OR instruments.ti,ab OR instruments.mp OR questionnaire.ti,ab OR questionnaire.mp OR questionnaires.ti,ab OR questionnaires.mp OR survey.ti,ab OR survey.mp OR surveys.ti,ab OR surveys.mp OR assess*.ti,ab OR assess*.mp OR coding scheme.ti,ab OR coding scheme.mp OR coding schemes.ti,ab OR codingscheme.ti,ab OR codingscheme.mp OR codingschemes.ti,ab OR codingschemes.mp OR rating.ti,ab OR rating.mp OR ratings.ti,ab OR ratings.mp OR selfreport.ti,ab OR selfreport.mp OR self report.ti,ab OR self report.mp OR selfreports.ti,ab OR selfreports.mp OR self reports.ti,ab OR self reports.mp OR "self report"/ OR *"Checklist"/ OR measure.ti,ab OR measure.mp OR measures.ti,ab OR measures.mp OR *"Observation"/ OR observation.ti,ab OR observation.mp OR observations.ti,ab OR observations.mp)

AND

(exp "intermethod comparison"/ OR exp "data collection method"/ OR exp "validation study"/ OR exp "feasibility study"/ OR exp "pilot study"/ OR exp "psychometry"/ OR exp "reproducibility"/ OR reproducib*.ti,ab OR "audit".ti,ab OR psychometr*.mp OR clinimetr*.ti,ab OR clinometr*.ti,ab OR exp "observer variation"/ OR "observer variation".ti,ab OR exp "discriminant analysis"/ OR exp "validity"/ OR reliab*.ti,ab OR valid*.ti,ab OR "coefficient".ti,ab OR "internal consistency".ti,ab OR (cronbach*.ti,ab AND ("alpha".ti,ab OR "alphas".ti,ab)) OR "item correlation".ti,ab OR "item correlations".ti,ab OR "item selection".ti,ab OR "item selections".ti,ab OR "item reduction".ti,ab OR "item reductions".ti,ab OR "agreement".ti,ab OR "precision".ti,ab OR "imprecision".ti,ab OR "precise values".ti,ab OR "test-retest".ti,ab OR ("test".ti,ab AND "retest".ti,ab) OR (reliab*.ti,ab AND ("test".ti,ab OR "retest".ti,ab)) OR "stability".ti,ab OR "interrater".ti,ab OR "inter-rater".ti,ab OR "intrarater".ti,ab OR "intra-rater".ti,ab OR "intertester".ti,ab OR "inter-tester".ti,ab OR "intratester".ti,ab OR "intra-tester".ti,ab OR "interobeserver".ti,ab OR "inter-observer".ti,ab OR "intraobserver".ti,ab OR "intra-observer".ti,ab OR "intertechnician".ti,ab OR "inter-technician".ti,ab OR "intratechnician".ti,ab OR "intra-technician".ti,ab OR "interexaminer".ti,ab OR "inter-examiner".ti,ab OR "intraexaminer".ti,ab OR "intra-examiner".ti,ab OR "interassay".ti,ab OR "inter-assay".ti,ab OR "intraassay".ti,ab OR "intra-assay".ti,ab OR "interindividual".ti,ab OR "inter-individual".ti,ab OR "intraindividual".ti,ab OR "intra-individual".ti,ab OR "interparticipant".ti,ab OR "inter-participant".ti,ab OR "intraparticipant".ti,ab OR "intra-participant".ti,ab OR "kappa".ti,ab OR "kappas".ti,ab OR "coefficient of variation".ti,ab OR repeatab*.ti,ab OR (replicab*.ti,ab OR "repeated".ti,ab AND ("measure".ti,ab OR "measures".ti,ab OR "findings".ti,ab OR "result".ti,ab OR "results".ti,ab OR "test".ti,ab OR "tests".ti,ab)) OR generaliza*.ti,ab OR generalisa*.ti,ab OR "concordance".ti,ab OR ("intraclass".ti,ab AND correlation*.ti,ab) OR "discriminative".ti,ab OR "known group".ti,ab OR "factor analysis".ti,ab OR "factor analyses".ti,ab OR "factor structure".ti,ab OR "factor structures".ti,ab OR "dimensionality".ti,ab OR subscale*.ti,ab OR "multitrait scaling analysis".ti,ab OR "multitrait scaling analyses".ti,ab OR "item discriminant".ti,ab OR "interscale correlation".ti,ab OR "interscale correlations".ti,ab OR ("error".ti,ab OR "errors".ti,ab AND (measure*.ti,ab OR correlat*.ti,ab OR evaluat*.ti,ab OR "accuracy".ti,ab OR "accurate".ti,ab OR "precision".ti,ab OR "mean".ti,ab)) OR "individual variability".ti,ab OR "interval variability".ti,ab OR "rate variability".ti,ab OR "variability analysis".ti,ab OR ("uncertainty".ti,ab AND ("measurement".ti,ab OR "measuring".ti,ab)) OR "standard error of measurement".ti,ab OR sensitiv*.ti,ab OR responsive*.ti,ab OR ("limit".ti,ab AND "detection".ti,ab) OR "minimal detectable concentration".ti,ab OR interpretab*.ti,ab OR (small*.ti,ab AND ("real".ti,ab OR "detectable".ti,ab) AND ("change".ti,ab OR "difference".ti,ab)) OR "meaningful change".ti,ab OR "minimal important change".ti,ab OR "minimal important difference".ti,ab OR "minimally important change".ti,ab OR "minimally important difference".ti,ab OR "minimal detectable change".ti,ab OR "minimal detectable difference".ti,ab OR "minimally detectable change".ti,ab OR "minimally detectable difference".ti,ab OR "minimal real change".ti,ab OR "minimal real difference".ti,ab OR "minimally real change".ti,ab OR "minimally real difference".ti,ab OR "ceiling effect".ti,ab OR "floor effect".ti,ab OR "item response model".ti,ab OR "irt".ti,ab OR "rasch".ti,ab OR "differential item functioning".ti,ab OR "dif".ti,ab OR "computer adaptive testing".ti,ab OR "item bank".ti,ab OR "cross-cultural equivalence".ti,ab OR exp Comparative Study/ OR "Outcome assessment"/ OR outcome assessment.mp OR outcome measure*.mp OR exp "Health Status Indicators"/ OR homogeneity.ti,ab OR homogeneous.ti,ab)

NOT

("editorial"/ OR "letter"/ OR conference abstract.pt OR conference review.pt) NOT (exp "Animals"/ NOT exp "humans"/)

### Cochrane

((("Decision Making" OR decision making OR decisionmaking) AND ("doctor patient relation" OR "nurse patient relationship" OR ((Patient) AND (provider OR physician OR professional OR doctor) AND (relation OR relations OR contact OR communication OR interaction OR interactions)) OR ((Patient) AND (provider OR physician OR professional OR doctor) AND (relation OR relations OR contact OR communication OR interaction OR interactions)) OR "Patient participation" OR Patient Participation OR patients participation OR patient's participation OR patient involvement OR patients involvement OR patient's involvement OR consultation* OR encounter)) OR shared decision OR shared decisions OR shared decisionmaking OR SDM OR Shared medical decision OR Shared treatment decision OR Shared medical decisions OR Shared treatment decisions OR Shared clinical decision OR Shared clinical decisions)

AND

("Health Care Survey" OR "Outcome Assessment" OR "Questionnaire" OR scale OR scale OR scales OR scales OR instrument OR instrument OR instruments OR instruments OR questionnaire OR questionnaire OR questionnaires OR questionnaires OR survey OR survey OR surveys OR surveys OR assess* OR assess* OR coding scheme OR coding scheme OR coding schemes OR codingscheme OR codingscheme OR codingschemes OR codingschemes OR rating OR rating OR ratings OR ratings OR selfreport OR selfreport OR self report OR self report OR selfreports OR selfreports OR self reports OR self reports OR "self report" OR "Checklist" OR measure OR measure OR measures OR measures OR "Observation" OR observation OR observation OR observations OR observations)

AND

("intermethod comparison" OR "data collection method" OR "validation study" OR "feasibility study" OR "pilot study" OR "psychometry" OR "reproducibility" OR reproducib* OR "audit" OR psychometr* OR clinimetr* OR clinometr* OR "observer variation" OR "observer variation" OR "discriminant analysis" OR "validity" OR reliab* OR valid* OR "coefficient" OR "internal consistency" OR (cronbach* AND ("alpha" OR "alphas")) OR "item correlation" OR "item correlations" OR "item selection" OR "item selections" OR "item reduction" OR "item reductions" OR "agreement" OR "precision" OR "imprecision" OR "precise values" OR "test-retest" OR ("test" AND "retest") OR (reliab* AND ("test" OR "retest")) OR "stability" OR "interrater" OR "inter-rater" OR "intrarater" OR "intra-rater" OR "intertester" OR "inter-tester" OR "intratester" OR "intra-tester" OR "interobeserver" OR "inter-observer" OR "intraobserver" OR "intra-observer" OR "intertechnician" OR "inter-technician" OR "intratechnician" OR "intra-technician" OR "interexaminer" OR "inter-examiner" OR "intraexaminer" OR "intra-examiner" OR "interassay" OR "inter-assay" OR "intraassay" OR "intra-assay" OR "interindividual" OR "inter-individual" OR "intraindividual" OR "intra-individual" OR "interparticipant" OR "inter-participant" OR "intraparticipant" OR "intra-participant" OR "kappa" OR "kappas" OR "coefficient of variation" OR repeatab* OR (replicab* OR "repeated" AND ("measure" OR "measures" OR "findings" OR "result" OR "results" OR "test" OR "tests")) OR generaliza* OR generalisa* OR "concordance" OR ("intraclass" AND correlation*) OR "discriminative" OR "known group" OR "factor analysis" OR "factor analyses" OR "factor structure" OR "factor structures" OR "dimensionality" OR subscale* OR "multitrait scaling analysis" OR "multitrait scaling analyses" OR "item discriminant" OR "interscale correlation" OR "interscale correlations" OR ("error" OR "errors" AND (measure* OR correlat* OR evaluat* OR "accuracy" OR "accurate" OR "precision" OR "mean")) OR "individual variability" OR "interval variability" OR "rate variability" OR "variability analysis" OR ("uncertainty" AND ("measurement" OR "measuring")) OR "standard error of measurement" OR sensitiv* OR responsive* OR ("limit" AND "detection") OR "minimal detectable concentration" OR interpretab* OR (small* AND ("real" OR "detectable") AND ("change" OR "difference")) OR "meaningful change" OR "minimal important change" OR "minimal important difference" OR "minimally important change" OR "minimally important difference" OR "minimal detectable change" OR "minimal detectable difference" OR "minimally detectable change" OR "minimally detectable difference" OR "minimal real change" OR "minimal real difference" OR "minimally real change" OR "minimally real difference" OR "ceiling effect" OR "floor effect" OR "item response model" OR "irt" OR "rasch" OR "differential item functioning" OR "dif" OR "computer adaptive testing" OR "item bank" OR "cross-cultural equivalence" OR Comparative Study OR "Outcome assessment" OR outcome assessment OR outcome measure* OR "Health Status Indicators" OR homogeneity OR homogeneous)

### PsycINFO

TI(((("Decision Making" OR decision making OR decisionmaking) AND ("doctor patient relation" OR "nurse patient relationship" OR ((Patient) AND (provider OR physician OR professional OR doctor) AND (relation OR relations OR contact OR communication OR interaction OR interactions)) OR ((Patient) AND (provider OR physician OR professional OR doctor) AND (relation OR relations OR contact OR communication OR interaction OR interactions)) OR "Patient participation" OR Patient Participation OR patients participation OR patient's participation OR patient involvement OR patients involvement OR patient's involvement OR consultation* OR encounter)) OR shared decision OR shared decisions OR shared decisionmaking OR SDM OR Shared medical decision OR Shared treatment decision OR Shared medical decisions OR Shared treatment decisions OR Shared clinical decision OR Shared clinical decisions)

AND

("Health Care Survey" OR "Outcome Assessment" OR "Questionnaire" OR scale OR scale OR scales OR scales OR instrument OR instrument OR instruments OR instruments OR questionnaire OR questionnaire OR questionnaires OR questionnaires OR survey OR survey OR surveys OR surveys OR assess* OR assess* OR coding scheme OR coding scheme OR coding schemes OR codingscheme OR codingscheme OR codingschemes OR codingschemes OR rating OR rating OR ratings OR ratings OR selfreport OR selfreport OR self report OR self report OR selfreports OR selfreports OR self reports OR self reports OR "self report" OR "Checklist" OR measure OR measure OR measures OR measures OR "Observation" OR observation OR observation OR observations OR observations)

AND

("intermethod comparison" OR "data collection method" OR "validation study" OR "feasibility study" OR "pilot study" OR "psychometry" OR "reproducibility" OR reproducib* OR "audit" OR psychometr* OR clinimetr* OR clinometr* OR "observer variation" OR "observer variation" OR "discriminant analysis" OR "validity" OR reliab* OR valid* OR "coefficient" OR "internal consistency" OR (cronbach* AND ("alpha" OR "alphas")) OR "item correlation" OR "item correlations" OR "item selection" OR "item selections" OR "item reduction" OR "item reductions" OR "agreement" OR "precision" OR "imprecision" OR "precise values" OR "test-retest" OR ("test" AND "retest") OR (reliab* AND ("test" OR "retest")) OR "stability" OR "interrater" OR "inter-rater" OR "intrarater" OR "intra-rater" OR "intertester" OR "inter-tester" OR "intratester" OR "intra-tester" OR "interobeserver" OR "inter-observer" OR "intraobserver" OR "intra-observer" OR "intertechnician" OR "inter-technician" OR "intratechnician" OR "intra-technician" OR "interexaminer" OR "inter-examiner" OR "intraexaminer" OR "intra-examiner" OR "interassay" OR "inter-assay" OR "intraassay" OR "intra-assay" OR "interindividual" OR "inter-individual" OR "intraindividual" OR "intra-individual" OR "interparticipant" OR "inter-participant" OR "intraparticipant" OR "intra-participant" OR "kappa" OR "kappas" OR "coefficient of variation" OR repeatab* OR (replicab* OR "repeated" AND ("measure" OR "measures" OR "findings" OR "result" OR "results" OR "test" OR "tests")) OR generaliza* OR generalisa* OR "concordance" OR ("intraclass" AND correlation*) OR "discriminative" OR "known group" OR "factor analysis" OR "factor analyses" OR "factor structure" OR "factor structures" OR "dimensionality" OR subscale* OR "multitrait scaling analysis" OR "multitrait scaling analyses" OR "item discriminant" OR "interscale correlation" OR "interscale correlations" OR ("error" OR "errors" AND (measure* OR correlat* OR evaluat* OR "accuracy" OR "accurate" OR "precision" OR "mean")) OR "individual variability" OR "interval variability" OR "rate variability" OR "variability analysis" OR ("uncertainty" AND ("measurement" OR "measuring")) OR "standard error of measurement" OR sensitiv* OR responsive* OR ("limit" AND "detection") OR "minimal detectable concentration" OR interpretab* OR (small* AND ("real" OR "detectable") AND ("change" OR "difference")) OR "meaningful change" OR "minimal important change" OR "minimal important difference" OR "minimally important change" OR "minimally important difference" OR "minimal detectable change" OR "minimal detectable difference" OR "minimally detectable change" OR "minimally detectable difference" OR "minimal real change" OR "minimal real difference" OR "minimally real change" OR "minimally real difference" OR "ceiling effect" OR "floor effect" OR "item response model" OR "irt" OR "rasch" OR "differential item functioning" OR "dif" OR "computer adaptive testing" OR "item bank" OR "cross-cultural equivalence" OR Comparative Study OR "Outcome assessment" OR outcome assessment OR outcome measure* OR "Health Status Indicators" OR homogeneity OR homogeneous))

OR

SU(((("Decision Making" OR decision making OR decisionmaking) AND ("doctor patient relation" OR "nurse patient relationship" OR ((Patient) AND (provider OR physician OR professional OR doctor) AND (relation OR relations OR contact OR communication OR interaction OR interactions)) OR ((Patient) AND (provider OR physician OR professional OR doctor) AND (relation OR relations OR contact OR communication OR interaction OR interactions)) OR "Patient participation" OR Patient Participation OR patients participation OR patient's participation OR patient involvement OR patients involvement OR patient's involvement OR consultation* OR encounter)) OR shared decision OR shared decisions OR shared decisionmaking OR SDM OR Shared medical decision OR Shared treatment decision OR Shared medical decisions OR Shared treatment decisions OR Shared clinical decision OR Shared clinical decisions)

AND

("Health Care Survey" OR "Outcome Assessment" OR "Questionnaire" OR scale OR scale OR scales OR scales OR instrument OR instrument OR instruments OR instruments OR questionnaire OR questionnaire OR questionnaires OR questionnaires OR survey OR survey OR surveys OR surveys OR assess* OR assess* OR coding scheme OR coding scheme OR coding schemes OR codingscheme OR codingscheme OR codingschemes OR codingschemes OR rating OR rating OR ratings OR ratings OR selfreport OR selfreport OR self report OR self report OR selfreports OR selfreports OR self reports OR self reports OR "self report" OR "Checklist" OR measure OR measure OR measures OR measures OR "Observation" OR observation OR observation OR observations OR observations)

AND

("intermethod comparison" OR "data collection method" OR "validation study" OR "feasibility study" OR "pilot study" OR "psychometry" OR "reproducibility" OR reproducib* OR "audit" OR psychometr* OR clinimetr* OR clinometr* OR "observer variation" OR "observer variation" OR "discriminant analysis" OR "validity" OR reliab* OR valid* OR "coefficient" OR "internal consistency" OR (cronbach* AND ("alpha" OR "alphas")) OR "item correlation" OR "item correlations" OR "item selection" OR "item selections" OR "item reduction" OR "item reductions" OR "agreement" OR "precision" OR "imprecision" OR "precise values" OR "test-retest" OR ("test" AND "retest") OR (reliab* AND ("test" OR "retest")) OR "stability" OR "interrater" OR "inter-rater" OR "intrarater" OR "intra-rater" OR "intertester" OR "inter-tester" OR "intratester" OR "intra-tester" OR "interobeserver" OR "inter-observer" OR "intraobserver" OR "intra-observer" OR "intertechnician" OR "inter-technician" OR "intratechnician" OR "intra-technician" OR "interexaminer" OR "inter-examiner" OR "intraexaminer" OR "intra-examiner" OR "interassay" OR "inter-assay" OR "intraassay" OR "intra-assay" OR "interindividual" OR "inter-individual" OR "intraindividual" OR "intra-individual" OR "interparticipant" OR "inter-participant" OR "intraparticipant" OR "intra-participant" OR "kappa" OR "kappas" OR "coefficient of variation" OR repeatab* OR (replicab* OR "repeated" AND ("measure" OR "measures" OR "findings" OR "result" OR "results" OR "test" OR "tests")) OR generaliza* OR generalisa* OR "concordance" OR ("intraclass" AND correlation*) OR "discriminative" OR "known group" OR "factor analysis" OR "factor analyses" OR "factor structure" OR "factor structures" OR "dimensionality" OR subscale* OR "multitrait scaling analysis" OR "multitrait scaling analyses" OR "item discriminant" OR "interscale correlation" OR "interscale correlations" OR ("error" OR "errors" AND (measure* OR correlat* OR evaluat* OR "accuracy" OR "accurate" OR "precision" OR "mean")) OR "individual variability" OR "interval variability" OR "rate variability" OR "variability analysis" OR ("uncertainty" AND ("measurement" OR "measuring")) OR "standard error of measurement" OR sensitiv* OR responsive* OR ("limit" AND "detection") OR "minimal detectable concentration" OR interpretab* OR (small* AND ("real" OR "detectable") AND ("change" OR "difference")) OR "meaningful change" OR "minimal important change" OR "minimal important difference" OR "minimally important change" OR "minimally important difference" OR "minimal detectable change" OR "minimal detectable difference" OR "minimally detectable change" OR "minimally detectable difference" OR "minimal real change" OR "minimal real difference" OR "minimally real change" OR "minimally real difference" OR "ceiling effect" OR "floor effect" OR "item response model" OR "irt" OR "rasch" OR "differential item functioning" OR "dif" OR "computer adaptive testing" OR "item bank" OR "cross-cultural equivalence" OR Comparative Study OR "Outcome assessment" OR outcome assessment OR outcome measure* OR "Health Status Indicators" OR homogeneity OR homogeneous))

OR

MA(((("Decision Making" OR decision making OR decisionmaking) AND ("doctor patient relation" OR "nurse patient relationship" OR ((Patient) AND (provider OR physician OR professional OR doctor) AND (relation OR relations OR contact OR communication OR interaction OR interactions)) OR ((Patient) AND (provider OR physician OR professional OR doctor) AND (relation OR relations OR contact OR communication OR interaction OR interactions)) OR "Patient participation" OR Patient Participation OR patients participation OR patient's participation OR patient involvement OR patients involvement OR patient's involvement OR consultation* OR encounter)) OR shared decision OR shared decisions OR shared decisionmaking OR SDM OR Shared medical decision OR Shared treatment decision OR Shared medical decisions OR Shared treatment decisions OR Shared clinical decision OR Shared clinical decisions)

AND

("Health Care Survey" OR "Outcome Assessment" OR "Questionnaire" OR scale OR scale OR scales OR scales OR instrument OR instrument OR instruments OR instruments OR questionnaire OR questionnaire OR questionnaires OR questionnaires OR survey OR survey OR surveys OR surveys OR assess* OR assess* OR coding scheme OR coding scheme OR coding schemes OR codingscheme OR codingscheme OR codingschemes OR codingschemes OR rating OR rating OR ratings OR ratings OR selfreport OR selfreport OR self report OR self report OR selfreports OR selfreports OR self reports OR self reports OR "self report" OR "Checklist" OR measure OR measure OR measures OR measures OR "Observation" OR observation OR observation OR observations OR observations)

AND

("intermethod comparison" OR "data collection method" OR "validation study" OR "feasibility study" OR "pilot study" OR "psychometry" OR "reproducibility" OR reproducib* OR "audit" OR psychometr* OR clinimetr* OR clinometr* OR "observer variation" OR "observer variation" OR "discriminant analysis" OR "validity" OR reliab* OR valid* OR "coefficient" OR "internal consistency" OR (cronbach* AND ("alpha" OR "alphas")) OR "item correlation" OR "item correlations" OR "item selection" OR "item selections" OR "item reduction" OR "item reductions" OR "agreement" OR "precision" OR "imprecision" OR "precise values" OR "test-retest" OR ("test" AND "retest") OR (reliab* AND ("test" OR "retest")) OR "stability" OR "interrater" OR "inter-rater" OR "intrarater" OR "intra-rater" OR "intertester" OR "inter-tester" OR "intratester" OR "intra-tester" OR "interobeserver" OR "inter-observer" OR "intraobserver" OR "intra-observer" OR "intertechnician" OR "inter-technician" OR "intratechnician" OR "intra-technician" OR "interexaminer" OR "inter-examiner" OR "intraexaminer" OR "intra-examiner" OR "interassay" OR "inter-assay" OR "intraassay" OR "intra-assay" OR "interindividual" OR "inter-individual" OR "intraindividual" OR "intra-individual" OR "interparticipant" OR "inter-participant" OR "intraparticipant" OR "intra-participant" OR "kappa" OR "kappas" OR "coefficient of variation" OR repeatab* OR (replicab* OR "repeated" AND ("measure" OR "measures" OR "findings" OR "result" OR "results" OR "test" OR "tests")) OR generaliza* OR generalisa* OR "concordance" OR ("intraclass" AND correlation*) OR "discriminative" OR "known group" OR "factor analysis" OR "factor analyses" OR "factor structure" OR "factor structures" OR "dimensionality" OR subscale* OR "multitrait scaling analysis" OR "multitrait scaling analyses" OR "item discriminant" OR "interscale correlation" OR "interscale correlations" OR ("error" OR "errors" AND (measure* OR correlat* OR evaluat* OR "accuracy" OR "accurate" OR "precision" OR "mean")) OR "individual variability" OR "interval variability" OR "rate variability" OR "variability analysis" OR ("uncertainty" AND ("measurement" OR "measuring")) OR "standard error of measurement" OR sensitiv* OR responsive* OR ("limit" AND "detection") OR "minimal detectable concentration" OR interpretab* OR (small* AND ("real" OR "detectable") AND ("change" OR "difference")) OR "meaningful change" OR "minimal important change" OR "minimal important difference" OR "minimally important change" OR "minimally important difference" OR "minimal detectable change" OR "minimal detectable difference" OR "minimally detectable change" OR "minimally detectable difference" OR "minimal real change" OR "minimal real difference" OR "minimally real change" OR "minimally real difference" OR "ceiling effect" OR "floor effect" OR "item response model" OR "irt" OR "rasch" OR "differential item functioning" OR "dif" OR "computer adaptive testing" OR "item bank" OR "cross-cultural equivalence" OR Comparative Study OR "Outcome assessment" OR outcome assessment OR outcome measure* OR "Health Status Indicators" OR homogeneity OR homogeneous))

OR

(AB(((("Decision Making" OR decision making OR decisionmaking) AND ("doctor patient relation" OR "nurse patient relationship" OR ((Patient) AND (provider OR physician OR professional OR doctor) AND (relation OR relations OR contact OR communication OR interaction OR interactions)) OR ((Patient) AND (provider OR physician OR professional OR doctor) AND (relation OR relations OR contact OR communication OR interaction OR interactions)) OR "Patient participation" OR Patient Participation OR patients participation OR patient's participation OR patient involvement OR patients involvement OR patient's involvement OR consultation* OR encounter)) OR shared decision OR shared decisions OR shared decisionmaking OR SDM OR Shared medical decision OR Shared treatment decision OR Shared medical decisions OR Shared treatment decisions OR Shared clinical decision OR Shared clinical decisions)

AND

("Health Care Survey" OR "Outcome Assessment" OR "Questionnaire" OR scale OR scale OR scales OR scales OR instrument OR instrument OR instruments OR instruments OR questionnaire OR questionnaire OR questionnaires OR questionnaires OR survey OR survey OR surveys OR surveys OR assess* OR assess* OR coding scheme OR coding scheme OR coding schemes OR codingscheme OR codingscheme OR codingschemes OR codingschemes OR rating OR rating OR ratings OR ratings OR selfreport OR selfreport OR self report OR self report OR selfreports OR selfreports OR self reports OR self reports OR "self report" OR "Checklist" OR measure OR measure OR measures OR measures OR "Observation" OR observation OR observation OR observations OR observations)

AND

("intermethod comparison" OR "data collection method" OR "validation study" OR "feasibility study" OR "pilot study" OR "psychometry" OR "reproducibility" OR reproducib* OR "audit" OR psychometr* OR clinimetr* OR clinometr* OR "observer variation" OR "observer variation" OR "discriminant analysis" OR "validity" OR reliab* OR valid* OR "coefficient" OR "internal consistency" OR (cronbach* AND ("alpha" OR "alphas")) OR "item correlation" OR "item correlations" OR "item selection" OR "item selections" OR "item reduction" OR "item reductions" OR "agreement" OR "precision" OR "imprecision" OR "precise values" OR "test-retest" OR ("test" AND "retest") OR (reliab* AND ("test" OR "retest")) OR "stability" OR "interrater" OR "inter-rater" OR "intrarater" OR "intra-rater" OR "intertester" OR "inter-tester" OR "intratester" OR "intra-tester" OR "interobeserver" OR "inter-observer" OR "intraobserver" OR "intra-observer" OR "intertechnician" OR "inter-technician" OR "intratechnician" OR "intra-technician" OR "interexaminer" OR "inter-examiner" OR "intraexaminer" OR "intra-examiner" OR "interassay" OR "inter-assay" OR "intraassay" OR "intra-assay" OR "interindividual" OR "inter-individual" OR "intraindividual" OR "intra-individual" OR "interparticipant" OR "inter-participant" OR "intraparticipant" OR "intra-participant" OR "kappa" OR "kappas" OR "coefficient of variation" OR repeatab* OR (replicab* OR "repeated" AND ("measure" OR "measures" OR "findings" OR "result" OR "results" OR "test" OR "tests")) OR generaliza* OR generalisa* OR "concordance" OR ("intraclass" AND correlation*) OR "discriminative" OR "known group" OR "factor analysis" OR "factor analyses" OR "factor structure" OR "factor structures" OR "dimensionality" OR subscale* OR "multitrait scaling analysis" OR "multitrait scaling analyses" OR "item discriminant" OR "interscale correlation" OR "interscale correlations" OR ("error" OR "errors" AND (measure* OR correlat* OR evaluat* OR "accuracy" OR "accurate" OR "precision" OR "mean")) OR "individual variability" OR "interval variability" OR "rate variability" OR "variability analysis" OR ("uncertainty" AND ("measurement" OR "measuring")) OR "standard error of measurement" OR sensitiv* OR responsive* OR ("limit" AND "detection") OR "minimal detectable concentration" OR interpretab* OR (small* AND ("real" OR "detectable") AND ("change" OR "difference")) OR "meaningful change" OR "minimal important change" OR "minimal important difference" OR "minimally important change" OR "minimally important difference" OR "minimal detectable change" OR "minimal detectable difference" OR "minimally detectable change" OR "minimally detectable difference" OR "minimal real change" OR "minimal real difference" OR "minimally real change" OR "minimally real difference" OR "ceiling effect" OR "floor effect" OR "item response model" OR "irt" OR "rasch" OR "differential item functioning" OR "dif" OR "computer adaptive testing" OR "item bank" OR "cross-cultural equivalence" OR Comparative Study OR "Outcome assessment" OR outcome assessment OR outcome measure* OR "Health Status Indicators" OR homogeneity OR homogeneous))

AND

TI(((("Decision Making" OR decision making OR decisionmaking) AND ("doctor patient relation" OR "nurse patient relationship" OR ((Patient) AND (provider OR physician OR professional OR doctor) AND (relation OR relations OR contact OR communication OR interaction OR interactions)) OR ((Patient) AND (provider OR physician OR professional OR doctor) AND (relation OR relations OR contact OR communication OR interaction OR interactions)) OR "Patient participation" OR Patient Participation OR patients participation OR patient's participation OR patient involvement OR patients involvement OR patient's involvement OR consultation* OR encounter)) OR shared decision OR shared decisions OR shared decisionmaking OR SDM OR Shared medical decision OR Shared treatment decision OR Shared medical decisions OR Shared treatment decisions OR Shared clinical decision OR Shared clinical decisions

OR

"Health Care Survey" OR "Outcome Assessment" OR "Questionnaire" OR scale OR scale OR scales OR scales OR instrument OR instrument OR instruments OR instruments OR questionnaire OR questionnaire OR questionnaires OR questionnaires OR survey OR survey OR surveys OR surveys OR assess* OR assess* OR coding scheme OR coding scheme OR coding schemes OR codingscheme OR codingscheme OR codingschemes OR codingschemes OR rating OR rating OR ratings OR ratings OR selfreport OR selfreport OR self report OR self report OR selfreports OR selfreports OR self reports OR self reports OR "self report" OR "Checklist" OR measure OR measure OR measures OR measures OR "Observation" OR observation OR observation OR observations OR observations

OR

"intermethod comparison" OR "data collection method" OR "validation study" OR "feasibility study" OR "pilot study" OR "psychometry" OR "reproducibility" OR reproducib* OR "audit" OR psychometr* OR clinimetr* OR clinometr* OR "observer variation" OR "observer variation" OR "discriminant analysis" OR "validity" OR reliab* OR valid* OR "coefficient" OR "internal consistency" OR (cronbach* AND ("alpha" OR "alphas")) OR "item correlation" OR "item correlations" OR "item selection" OR "item selections" OR "item reduction" OR "item reductions" OR "agreement" OR "precision" OR "imprecision" OR "precise values" OR "test-retest" OR ("test" AND "retest") OR (reliab* AND ("test" OR "retest")) OR "stability" OR "interrater" OR "inter-rater" OR "intrarater" OR "intra-rater" OR "intertester" OR "inter-tester" OR "intratester" OR "intra-tester" OR "interobeserver" OR "inter-observer" OR "intraobserver" OR "intra-observer" OR "intertechnician" OR "inter-technician" OR "intratechnician" OR "intra-technician" OR "interexaminer" OR "inter-examiner" OR "intraexaminer" OR "intra-examiner" OR "interassay" OR "inter-assay" OR "intraassay" OR "intra-assay" OR "interindividual" OR "inter-individual" OR "intraindividual" OR "intra-individual" OR "interparticipant" OR "inter-participant" OR "intraparticipant" OR "intra-participant" OR "kappa" OR "kappas" OR "coefficient of variation" OR repeatab* OR (replicab* OR "repeated" AND ("measure" OR "measures" OR "findings" OR "result" OR "results" OR "test" OR "tests")) OR generaliza* OR generalisa* OR "concordance" OR ("intraclass" AND correlation*) OR "discriminative" OR "known group" OR "factor analysis" OR "factor analyses" OR "factor structure" OR "factor structures" OR "dimensionality" OR subscale* OR "multitrait scaling analysis" OR "multitrait scaling analyses" OR "item discriminant" OR "interscale correlation" OR "interscale correlations" OR ("error" OR "errors" AND (measure* OR correlat* OR evaluat* OR "accuracy" OR "accurate" OR "precision" OR "mean")) OR "individual variability" OR "interval variability" OR "rate variability" OR "variability analysis" OR ("uncertainty" AND ("measurement" OR "measuring")) OR "standard error of measurement" OR sensitiv* OR responsive* OR ("limit" AND "detection") OR "minimal detectable concentration" OR interpretab* OR (small* AND ("real" OR "detectable") AND ("change" OR "difference")) OR "meaningful change" OR "minimal important change" OR "minimal important difference" OR "minimally important change" OR "minimally important difference" OR "minimal detectable change" OR "minimal detectable difference" OR "minimally detectable change" OR "minimally detectable difference" OR "minimal real change" OR "minimal real difference" OR "minimally real change" OR "minimally real difference" OR "ceiling effect" OR "floor effect" OR "item response model" OR "irt" OR "rasch" OR "differential item functioning" OR "dif" OR "computer adaptive testing" OR "item bank" OR "cross-cultural equivalence" OR Comparative Study OR "Outcome assessment" OR outcome assessment OR outcome measure* OR "Health Status Indicators" OR homogeneity OR homogeneous)))

### Web of science

(TI = ((("Decision Making" OR decision making OR decisionmaking) AND ("doctor patient relation" OR "nurse patient relationship" OR ((Patient) AND (provider OR physician OR professional OR doctor) AND (relation OR relations OR contact OR communication OR interaction OR interactions)) OR ((Patient) AND (provider OR physician OR professional OR doctor) AND (relation OR relations OR contact OR communication OR interaction OR interactions)) OR "Patient participation" OR Patient Participation OR patients participation OR patient's participation OR patient involvement OR patients involvement OR patient's involvement OR consultation* OR encounter)) OR shared decision OR shared decisions OR shared decisionmaking OR SDM OR Shared medical decision OR Shared treatment decision OR Shared medical decisions OR Shared treatment decisions OR Shared clinical decision OR Shared clinical decisions)

AND

TI = ("Health Care Survey" OR "Outcome Assessment" OR "Questionnaire" OR scale OR scale OR scales OR scales OR instrument OR instrument OR instruments OR instruments OR questionnaire OR questionnaire OR questionnaires OR questionnaires OR survey OR survey OR surveys OR surveys OR assess* OR assess* OR coding scheme OR coding scheme OR coding schemes OR codingscheme OR codingscheme OR codingschemes OR codingschemes OR rating OR rating OR ratings OR ratings OR selfreport OR selfreport OR self report OR self report OR selfreports OR selfreports OR self reports OR self reports OR "self report" OR "Checklist" OR measure OR measure OR measures OR measures OR "Observation" OR observation OR observation OR observations OR observations)

AND

TS = ("intermethod comparison" OR "data collection method" OR "validation study" OR "feasibility study" OR "pilot study" OR "psychometry" OR "reproducibility" OR reproducib* OR "audit" OR psychometr* OR clinimetr* OR clinometr* OR "observer variation" OR "observer variation" OR "discriminant analysis" OR "validity" OR reliab* OR valid* OR "coefficient" OR "internal consistency" OR (cronbach* AND ("alpha" OR "alphas")) OR "item correlation" OR "item correlations" OR "item selection" OR "item selections" OR "item reduction" OR "item reductions" OR "agreement" OR "precision" OR "imprecision" OR "precise values" OR "test-retest" OR ("test" AND "retest") OR (reliab* AND ("test" OR "retest")) OR "stability" OR "interrater" OR "inter-rater" OR "intrarater" OR "intra-rater" OR "intertester" OR "inter-tester" OR "intratester" OR "intra-tester" OR "interobeserver" OR "inter-observer" OR "intraobserver" OR "intra-observer" OR "intertechnician" OR "inter-technician" OR "intratechnician" OR "intra-technician" OR "interexaminer" OR "inter-examiner" OR "intraexaminer" OR "intra-examiner" OR "interassay" OR "inter-assay" OR "intraassay" OR "intra-assay" OR "interindividual" OR "inter-individual" OR "intraindividual" OR "intra-individual" OR "interparticipant" OR "inter-participant" OR "intraparticipant" OR "intra-participant" OR "kappa" OR "kappas" OR "coefficient of variation" OR repeatab* OR (replicab* OR "repeated" AND ("measure" OR "measures" OR "findings" OR "result" OR "results" OR "test" OR "tests")) OR generaliza* OR generalisa* OR "concordance" OR ("intraclass" AND correlation*) OR "discriminative" OR "known group" OR "factor analysis" OR "factor analyses" OR "factor structure" OR "factor structures" OR "dimensionality" OR subscale* OR "multitrait scaling analysis" OR "multitrait scaling analyses" OR "item discriminant" OR "interscale correlation" OR "interscale correlations" OR ("error" OR "errors" AND (measure* OR correlat* OR evaluat* OR "accuracy" OR "accurate" OR "precision" OR "mean")) OR "individual variability" OR "interval variability" OR "rate variability" OR "variability analysis" OR ("uncertainty" AND ("measurement" OR "measuring")) OR "standard error of measurement" OR sensitiv* OR responsive* OR ("limit" AND "detection") OR "minimal detectable concentration" OR interpretab* OR (small* AND ("real" OR "detectable") AND ("change" OR "difference")) OR "meaningful change" OR "minimal important change" OR "minimal important difference" OR "minimally important change" OR "minimally important difference" OR "minimal detectable change" OR "minimal detectable difference" OR "minimally detectable change" OR "minimally detectable difference" OR "minimal real change" OR "minimal real difference" OR "minimally real change" OR "minimally real difference" OR "ceiling effect" OR "floor effect" OR "item response model" OR "irt" OR "rasch" OR "differential item functioning" OR "dif" OR "computer adaptive testing" OR "item bank" OR "cross-cultural equivalence" OR Comparative Study OR "Outcome assessment" OR outcome assessment OR outcome measure* OR "Health Status Indicators" OR homogeneity OR homogeneous))

OR

(TI = ((("Decision Making" OR decision making OR decisionmaking) AND ("doctor patient relation" OR "nurse patient relationship" OR ((Patient) AND (provider OR physician OR professional OR doctor) AND (relation OR relations OR contact OR communication OR interaction OR interactions)) OR ((Patient) AND (provider OR physician OR professional OR doctor) AND (relation OR relations OR contact OR communication OR interaction OR interactions)) OR "Patient participation" OR Patient Participation OR patients participation OR patient's participation OR patient involvement OR patients involvement OR patient's involvement OR consultation* OR encounter)) OR shared decision OR shared decisions OR shared decisionmaking OR SDM OR Shared medical decision OR Shared treatment decision OR Shared medical decisions OR Shared treatment decisions OR Shared clinical decision OR Shared clinical decisions)

AND

TS = ("Health Care Survey" OR "Outcome Assessment" OR "Questionnaire" OR scale OR scale OR scales OR scales OR instrument OR instrument OR instruments OR instruments OR questionnaire OR questionnaire OR questionnaires OR questionnaires OR survey OR survey OR surveys OR surveys OR assess* OR assess* OR coding scheme OR coding scheme OR coding schemes OR codingscheme OR codingscheme OR codingschemes OR codingschemes OR rating OR rating OR ratings OR ratings OR selfreport OR selfreport OR self report OR self report OR selfreports OR selfreports OR self reports OR self reports OR "self report" OR "Checklist" OR measure OR measure OR measures OR measures OR "Observation" OR observation OR observation OR observations OR observations)

AND

TI = ("intermethod comparison" OR "data collection method" OR "validation study" OR "feasibility study" OR "pilot study" OR "psychometry" OR "reproducibility" OR reproducib* OR "audit" OR psychometr* OR clinimetr* OR clinometr* OR "observer variation" OR "observer variation" OR "discriminant analysis" OR "validity" OR reliab* OR valid* OR "coefficient" OR "internal consistency" OR (cronbach* AND ("alpha" OR "alphas")) OR "item correlation" OR "item correlations" OR "item selection" OR "item selections" OR "item reduction" OR "item reductions" OR "agreement" OR "precision" OR "imprecision" OR "precise values" OR "test-retest" OR ("test" AND "retest") OR (reliab* AND ("test" OR "retest")) OR "stability" OR "interrater" OR "inter-rater" OR "intrarater" OR "intra-rater" OR "intertester" OR "inter-tester" OR "intratester" OR "intra-tester" OR "interobeserver" OR "inter-observer" OR "intraobserver" OR "intra-observer" OR "intertechnician" OR "inter-technician" OR "intratechnician" OR "intra-technician" OR "interexaminer" OR "inter-examiner" OR "intraexaminer" OR "intra-examiner" OR "interassay" OR "inter-assay" OR "intraassay" OR "intra-assay" OR "interindividual" OR "inter-individual" OR "intraindividual" OR "intra-individual" OR "interparticipant" OR "inter-participant" OR "intraparticipant" OR "intra-participant" OR "kappa" OR "kappas" OR "coefficient of variation" OR repeatab* OR (replicab* OR "repeated" AND ("measure" OR "measures" OR "findings" OR "result" OR "results" OR "test" OR "tests")) OR generaliza* OR generalisa* OR "concordance" OR ("intraclass" AND correlation*) OR "discriminative" OR "known group" OR "factor analysis" OR "factor analyses" OR "factor structure" OR "factor structures" OR "dimensionality" OR subscale* OR "multitrait scaling analysis" OR "multitrait scaling analyses" OR "item discriminant" OR "interscale correlation" OR "interscale correlations" OR ("error" OR "errors" AND (measure* OR correlat* OR evaluat* OR "accuracy" OR "accurate" OR "precision" OR "mean")) OR "individual variability" OR "interval variability" OR "rate variability" OR "variability analysis" OR ("uncertainty" AND ("measurement" OR "measuring")) OR "standard error of measurement" OR sensitiv* OR responsive* OR ("limit" AND "detection") OR "minimal detectable concentration" OR interpretab* OR (small* AND ("real" OR "detectable") AND ("change" OR "difference")) OR "meaningful change" OR "minimal important change" OR "minimal important difference" OR "minimally important change" OR "minimally important difference" OR "minimal detectable change" OR "minimal detectable difference" OR "minimally detectable change" OR "minimally detectable difference" OR "minimal real change" OR "minimal real difference" OR "minimally real change" OR "minimally real difference" OR "ceiling effect" OR "floor effect" OR "item response model" OR "irt" OR "rasch" OR "differential item functioning" OR "dif" OR "computer adaptive testing" OR "item bank" OR "cross-cultural equivalence" OR Comparative Study OR "Outcome assessment" OR outcome assessment OR outcome measure* OR "Health Status Indicators" OR homogeneity OR homogeneous))

OR

(TS = ((("Decision Making" OR decision making OR decisionmaking) AND ("doctor patient relation" OR "nurse patient relationship" OR ((Patient) AND (provider OR physician OR professional OR doctor) AND (relation OR relations OR contact OR communication OR interaction OR interactions)) OR ((Patient) AND (provider OR physician OR professional OR doctor) AND (relation OR relations OR contact OR communication OR interaction OR interactions)) OR "Patient participation" OR Patient Participation OR patients participation OR patient's participation OR patient involvement OR patients involvement OR patient's involvement OR consultation* OR encounter)) OR shared decision OR shared decisions OR shared decisionmaking OR SDM OR Shared medical decision OR Shared treatment decision OR Shared medical decisions OR Shared treatment decisions OR Shared clinical decision OR Shared clinical decisions)

AND

TI = ("Health Care Survey" OR "Outcome Assessment" OR "Questionnaire" OR scale OR scale OR scales OR scales OR instrument OR instrument OR instruments OR instruments OR questionnaire OR questionnaire OR questionnaires OR questionnaires OR survey OR survey OR surveys OR surveys OR assess* OR assess* OR coding scheme OR coding scheme OR coding schemes OR codingscheme OR codingscheme OR codingschemes OR codingschemes OR rating OR rating OR ratings OR ratings OR selfreport OR selfreport OR self report OR self report OR selfreports OR selfreports OR self reports OR self reports OR "self report" OR "Checklist" OR measure OR measure OR measures OR measures OR "Observation" OR observation OR observation OR observations OR observations)

AND

TI = ("intermethod comparison" OR "data collection method" OR "validation study" OR "feasibility study" OR "pilot study" OR "psychometry" OR "reproducibility" OR reproducib* OR "audit" OR psychometr* OR clinimetr* OR clinometr* OR "observer variation" OR "observer variation" OR "discriminant analysis" OR "validity" OR reliab* OR valid* OR "coefficient" OR "internal consistency" OR (cronbach* AND ("alpha" OR "alphas")) OR "item correlation" OR "item correlations" OR "item selection" OR "item selections" OR "item reduction" OR "item reductions" OR "agreement" OR "precision" OR "imprecision" OR "precise values" OR "test-retest" OR ("test" AND "retest") OR (reliab* AND ("test" OR "retest")) OR "stability" OR "interrater" OR "inter-rater" OR "intrarater" OR "intra-rater" OR "intertester" OR "inter-tester" OR "intratester" OR "intra-tester" OR "interobeserver" OR "inter-observer" OR "intraobserver" OR "intra-observer" OR "intertechnician" OR "inter-technician" OR "intratechnician" OR "intra-technician" OR "interexaminer" OR "inter-examiner" OR "intraexaminer" OR "intra-examiner" OR "interassay" OR "inter-assay" OR "intraassay" OR "intra-assay" OR "interindividual" OR "inter-individual" OR "intraindividual" OR "intra-individual" OR "interparticipant" OR "inter-participant" OR "intraparticipant" OR "intra-participant" OR "kappa" OR "kappas" OR "coefficient of variation" OR repeatab* OR (replicab* OR "repeated" AND ("measure" OR "measures" OR "findings" OR "result" OR "results" OR "test" OR "tests")) OR generaliza* OR generalisa* OR "concordance" OR ("intraclass" AND correlation*) OR "discriminative" OR "known group" OR "factor analysis" OR "factor analyses" OR "factor structure" OR "factor structures" OR "dimensionality" OR subscale* OR "multitrait scaling analysis" OR "multitrait scaling analyses" OR "item discriminant" OR "interscale correlation" OR "interscale correlations" OR ("error" OR "errors" AND (measure* OR correlat* OR evaluat* OR "accuracy" OR "accurate" OR "precision" OR "mean")) OR "individual variability" OR "interval variability" OR "rate variability" OR "variability analysis" OR ("uncertainty" AND ("measurement" OR "measuring")) OR "standard error of measurement" OR sensitiv* OR responsive* OR ("limit" AND "detection") OR "minimal detectable concentration" OR interpretab* OR (small* AND ("real" OR "detectable") AND ("change" OR "difference")) OR "meaningful change" OR "minimal important change" OR "minimal important difference" OR "minimally important change" OR "minimally important difference" OR "minimal detectable change" OR "minimal detectable difference" OR "minimally detectable change" OR "minimally detectable difference" OR "minimal real change" OR "minimal real difference" OR "minimally real change" OR "minimally real difference" OR "ceiling effect" OR "floor effect" OR "item response model" OR "irt" OR "rasch" OR "differential item functioning" OR "dif" OR "computer adaptive testing" OR "item bank" OR "cross-cultural equivalence" OR Comparative Study OR "Outcome assessment" OR outcome assessment OR outcome measure* OR "Health Status Indicators" OR homogeneity OR homogeneous))

### Academic Search Premier

TI(((("Decision Making" OR decision making OR decisionmaking) AND ("doctor patient relation" OR "nurse patient relationship" OR ((Patient) AND (provider OR physician OR professional OR doctor) AND (relation OR relations OR contact OR communication OR interaction OR interactions)) OR ((Patient) AND (provider OR physician OR professional OR doctor) AND (relation OR relations OR contact OR communication OR interaction OR interactions)) OR "Patient participation" OR Patient Participation OR patients participation OR patient's participation OR patient involvement OR patients involvement OR patient's involvement OR consultation* OR encounter)) OR shared decision OR shared decisions OR shared decisionmaking OR SDM OR Shared medical decision OR Shared treatment decision OR Shared medical decisions OR Shared treatment decisions OR Shared clinical decision OR Shared clinical decisions)

AND

("Health Care Survey" OR "Outcome Assessment" OR "Questionnaire" OR scale OR scale OR scales OR scales OR instrument OR instrument OR instruments OR instruments OR questionnaire OR questionnaire OR questionnaires OR questionnaires OR survey OR survey OR surveys OR surveys OR assess* OR assess* OR coding scheme OR coding scheme OR coding schemes OR codingscheme OR codingscheme OR codingschemes OR codingschemes OR rating OR rating OR ratings OR ratings OR selfreport OR selfreport OR self report OR self report OR selfreports OR selfreports OR self reports OR self reports OR "self report" OR "Checklist" OR measure OR measure OR measures OR measures OR "Observation" OR observation OR observation OR observations OR observations)

AND

("intermethod comparison" OR "data collection method" OR "validation study" OR "feasibility study" OR "pilot study" OR "psychometry" OR "reproducibility" OR reproducib* OR "audit" OR psychometr* OR clinimetr* OR clinometr* OR "observer variation" OR "observer variation" OR "discriminant analysis" OR "validity" OR reliab* OR valid* OR "coefficient" OR "internal consistency" OR (cronbach* AND ("alpha" OR "alphas")) OR "item correlation" OR "item correlations" OR "item selection" OR "item selections" OR "item reduction" OR "item reductions" OR "agreement" OR "precision" OR "imprecision" OR "precise values" OR "test-retest" OR ("test" AND "retest") OR (reliab* AND ("test" OR "retest")) OR "stability" OR "interrater" OR "inter-rater" OR "intrarater" OR "intra-rater" OR "intertester" OR "inter-tester" OR "intratester" OR "intra-tester" OR "interobeserver" OR "inter-observer" OR "intraobserver" OR "intra-observer" OR "intertechnician" OR "inter-technician" OR "intratechnician" OR "intra-technician" OR "interexaminer" OR "inter-examiner" OR "intraexaminer" OR "intra-examiner" OR "interassay" OR "inter-assay" OR "intraassay" OR "intra-assay" OR "interindividual" OR "inter-individual" OR "intraindividual" OR "intra-individual" OR "interparticipant" OR "inter-participant" OR "intraparticipant" OR "intra-participant" OR "kappa" OR "kappas" OR "coefficient of variation" OR repeatab* OR (replicab* OR "repeated" AND ("measure" OR "measures" OR "findings" OR "result" OR "results" OR "test" OR "tests")) OR generaliza* OR generalisa* OR "concordance" OR ("intraclass" AND correlation*) OR "discriminative" OR "known group" OR "factor analysis" OR "factor analyses" OR "factor structure" OR "factor structures" OR "dimensionality" OR subscale* OR "multitrait scaling analysis" OR "multitrait scaling analyses" OR "item discriminant" OR "interscale correlation" OR "interscale correlations" OR ("error" OR "errors" AND (measure* OR correlat* OR evaluat* OR "accuracy" OR "accurate" OR "precision" OR "mean")) OR "individual variability" OR "interval variability" OR "rate variability" OR "variability analysis" OR ("uncertainty" AND ("measurement" OR "measuring")) OR "standard error of measurement" OR sensitiv* OR responsive* OR ("limit" AND "detection") OR "minimal detectable concentration" OR interpretab* OR (small* AND ("real" OR "detectable") AND ("change" OR "difference")) OR "meaningful change" OR "minimal important change" OR "minimal important difference" OR "minimally important change" OR "minimally important difference" OR "minimal detectable change" OR "minimal detectable difference" OR "minimally detectable change" OR "minimally detectable difference" OR "minimal real change" OR "minimal real difference" OR "minimally real change" OR "minimally real difference" OR "ceiling effect" OR "floor effect" OR "item response model" OR "irt" OR "rasch" OR "differential item functioning" OR "dif" OR "computer adaptive testing" OR "item bank" OR "cross-cultural equivalence" OR Comparative Study OR "Outcome assessment" OR outcome assessment OR outcome measure* OR "Health Status Indicators" OR homogeneity OR homogeneous))

OR

SU(((("Decision Making" OR decision making OR decisionmaking) AND ("doctor patient relation" OR "nurse patient relationship" OR ((Patient) AND (provider OR physician OR professional OR doctor) AND (relation OR relations OR contact OR communication OR interaction OR interactions)) OR ((Patient) AND (provider OR physician OR professional OR doctor) AND (relation OR relations OR contact OR communication OR interaction OR interactions)) OR "Patient participation" OR Patient Participation OR patients participation OR patient's participation OR patient involvement OR patients involvement OR patient's involvement OR consultation* OR encounter)) OR shared decision OR shared decisions OR shared decisionmaking OR SDM OR Shared medical decision OR Shared treatment decision OR Shared medical decisions OR Shared treatment decisions OR Shared clinical decision OR Shared clinical decisions)

AND

("Health Care Survey" OR "Outcome Assessment" OR "Questionnaire" OR scale OR scale OR scales OR scales OR instrument OR instrument OR instruments OR instruments OR questionnaire OR questionnaire OR questionnaires OR questionnaires OR survey OR survey OR surveys OR surveys OR assess* OR assess* OR coding scheme OR coding scheme OR coding schemes OR codingscheme OR codingscheme OR codingschemes OR codingschemes OR rating OR rating OR ratings OR ratings OR selfreport OR selfreport OR self report OR self report OR selfreports OR selfreports OR self reports OR self reports OR "self report" OR "Checklist" OR measure OR measure OR measures OR measures OR "Observation" OR observation OR observation OR observations OR observations)

AND

("intermethod comparison" OR "data collection method" OR "validation study" OR "feasibility study" OR "pilot study" OR "psychometry" OR "reproducibility" OR reproducib* OR "audit" OR psychometr* OR clinimetr* OR clinometr* OR "observer variation" OR "observer variation" OR "discriminant analysis" OR "validity" OR reliab* OR valid* OR "coefficient" OR "internal consistency" OR (cronbach* AND ("alpha" OR "alphas")) OR "item correlation" OR "item correlations" OR "item selection" OR "item selections" OR "item reduction" OR "item reductions" OR "agreement" OR "precision" OR "imprecision" OR "precise values" OR "test-retest" OR ("test" AND "retest") OR (reliab* AND ("test" OR "retest")) OR "stability" OR "interrater" OR "inter-rater" OR "intrarater" OR "intra-rater" OR "intertester" OR "inter-tester" OR "intratester" OR "intra-tester" OR "interobeserver" OR "inter-observer" OR "intraobserver" OR "intra-observer" OR "intertechnician" OR "inter-technician" OR "intratechnician" OR "intra-technician" OR "interexaminer" OR "inter-examiner" OR "intraexaminer" OR "intra-examiner" OR "interassay" OR "inter-assay" OR "intraassay" OR "intra-assay" OR "interindividual" OR "inter-individual" OR "intraindividual" OR "intra-individual" OR "interparticipant" OR "inter-participant" OR "intraparticipant" OR "intra-participant" OR "kappa" OR "kappas" OR "coefficient of variation" OR repeatab* OR (replicab* OR "repeated" AND ("measure" OR "measures" OR "findings" OR "result" OR "results" OR "test" OR "tests")) OR generaliza* OR generalisa* OR "concordance" OR ("intraclass" AND correlation*) OR "discriminative" OR "known group" OR "factor analysis" OR "factor analyses" OR "factor structure" OR "factor structures" OR "dimensionality" OR subscale* OR "multitrait scaling analysis" OR "multitrait scaling analyses" OR "item discriminant" OR "interscale correlation" OR "interscale correlations" OR ("error" OR "errors" AND (measure* OR correlat* OR evaluat* OR "accuracy" OR "accurate" OR "precision" OR "mean")) OR "individual variability" OR "interval variability" OR "rate variability" OR "variability analysis" OR ("uncertainty" AND ("measurement" OR "measuring")) OR "standard error of measurement" OR sensitiv* OR responsive* OR ("limit" AND "detection") OR "minimal detectable concentration" OR interpretab* OR (small* AND ("real" OR "detectable") AND ("change" OR "difference")) OR "meaningful change" OR "minimal important change" OR "minimal important difference" OR "minimally important change" OR "minimally important difference" OR "minimal detectable change" OR "minimal detectable difference" OR "minimally detectable change" OR "minimally detectable difference" OR "minimal real change" OR "minimal real difference" OR "minimally real change" OR "minimally real difference" OR "ceiling effect" OR "floor effect" OR "item response model" OR "irt" OR "rasch" OR "differential item functioning" OR "dif" OR "computer adaptive testing" OR "item bank" OR "cross-cultural equivalence" OR Comparative Study OR "Outcome assessment" OR outcome assessment OR outcome measure* OR "Health Status Indicators" OR homogeneity OR homogeneous))

OR

KW(((("Decision Making" OR decision making OR decisionmaking) AND ("doctor patient relation" OR "nurse patient relationship" OR ((Patient) AND (provider OR physician OR professional OR doctor) AND (relation OR relations OR contact OR communication OR interaction OR interactions)) OR ((Patient) AND (provider OR physician OR professional OR doctor) AND (relation OR relations OR contact OR communication OR interaction OR interactions)) OR "Patient participation" OR Patient Participation OR patients participation OR patient's participation OR patient involvement OR patients involvement OR patient's involvement OR consultation* OR encounter)) OR shared decision OR shared decisions OR shared decisionmaking OR SDM OR Shared medical decision OR Shared treatment decision OR Shared medical decisions OR Shared treatment decisions OR Shared clinical decision OR Shared clinical decisions)

AND

("Health Care Survey" OR "Outcome Assessment" OR "Questionnaire" OR scale OR scale OR scales OR scales OR instrument OR instrument OR instruments OR instruments OR questionnaire OR questionnaire OR questionnaires OR questionnaires OR survey OR survey OR surveys OR surveys OR assess* OR assess* OR coding scheme OR coding scheme OR coding schemes OR codingscheme OR codingscheme OR codingschemes OR codingschemes OR rating OR rating OR ratings OR ratings OR selfreport OR selfreport OR self report OR self report OR selfreports OR selfreports OR self reports OR self reports OR "self report" OR "Checklist" OR measure OR measure OR measures OR measures OR "Observation" OR observation OR observation OR observations OR observations)

AND

("intermethod comparison" OR "data collection method" OR "validation study" OR "feasibility study" OR "pilot study" OR "psychometry" OR "reproducibility" OR reproducib* OR "audit" OR psychometr* OR clinimetr* OR clinometr* OR "observer variation" OR "observer variation" OR "discriminant analysis" OR "validity" OR reliab* OR valid* OR "coefficient" OR "internal consistency" OR (cronbach* AND ("alpha" OR "alphas")) OR "item correlation" OR "item correlations" OR "item selection" OR "item selections" OR "item reduction" OR "item reductions" OR "agreement" OR "precision" OR "imprecision" OR "precise values" OR "test-retest" OR ("test" AND "retest") OR (reliab* AND ("test" OR "retest")) OR "stability" OR "interrater" OR "inter-rater" OR "intrarater" OR "intra-rater" OR "intertester" OR "inter-tester" OR "intratester" OR "intra-tester" OR "interobeserver" OR "inter-observer" OR "intraobserver" OR "intra-observer" OR "intertechnician" OR "inter-technician" OR "intratechnician" OR "intra-technician" OR "interexaminer" OR "inter-examiner" OR "intraexaminer" OR "intra-examiner" OR "interassay" OR "inter-assay" OR "intraassay" OR "intra-assay" OR "interindividual" OR "inter-individual" OR "intraindividual" OR "intra-individual" OR "interparticipant" OR "inter-participant" OR "intraparticipant" OR "intra-participant" OR "kappa" OR "kappas" OR "coefficient of variation" OR repeatab* OR (replicab* OR "repeated" AND ("measure" OR "measures" OR "findings" OR "result" OR "results" OR "test" OR "tests")) OR generaliza* OR generalisa* OR "concordance" OR ("intraclass" AND correlation*) OR "discriminative" OR "known group" OR "factor analysis" OR "factor analyses" OR "factor structure" OR "factor structures" OR "dimensionality" OR subscale* OR "multitrait scaling analysis" OR "multitrait scaling analyses" OR "item discriminant" OR "interscale correlation" OR "interscale correlations" OR ("error" OR "errors" AND (measure* OR correlat* OR evaluat* OR "accuracy" OR "accurate" OR "precision" OR "mean")) OR "individual variability" OR "interval variability" OR "rate variability" OR "variability analysis" OR ("uncertainty" AND ("measurement" OR "measuring")) OR "standard error of measurement" OR sensitiv* OR responsive* OR ("limit" AND "detection") OR "minimal detectable concentration" OR interpretab* OR (small* AND ("real" OR "detectable") AND ("change" OR "difference")) OR "meaningful change" OR "minimal important change" OR "minimal important difference" OR "minimally important change" OR "minimally important difference" OR "minimal detectable change" OR "minimal detectable difference" OR "minimally detectable change" OR "minimally detectable difference" OR "minimal real change" OR "minimal real difference" OR "minimally real change" OR "minimally real difference" OR "ceiling effect" OR "floor effect" OR "item response model" OR "irt" OR "rasch" OR "differential item functioning" OR "dif" OR "computer adaptive testing" OR "item bank" OR "cross-cultural equivalence" OR Comparative Study OR "Outcome assessment" OR outcome assessment OR outcome measure* OR "Health Status Indicators" OR homogeneity OR homogeneous))

## Supporting information

S1 Table Extracted data. (XLSX)

S2 Table Methodological quality and quality of measurement properties of each article per measurement property and instrument version. Note: Measurement error is not presented as one of the measurement properties because it has not been evaluated in any of the articles. M = result of the methodological quality appraisal with a score on the 4-point rating scale based on the COSMIN: poor, fair, good, excellent R = result of the quality of measurement property appraisal with three possible categories: + = positive,? = inconclusive,— = negative; n.i. = not investigated, n.a. = not applicable, m = missing, CFA = confirmative factor analysis **Reference [43] also presents results of the development and validation for the SMDMQ (Taiwanese), however the results seem the exact same as presented in[42]; reference [43] was therefore left out in the data extraction and analysis and also not included in the number of included articles. ** The negative score is based on hypotheses that were not confirmed because correlations were high instead of medium-sized, thus, hypotheses testing actually showed that there is a strong relationship with instruments measuring the same construct.
(DOCX)
