# Supplementary material for: The quality of instruments to assess the process of shared decision making: A systematic review
Source: PLoS One. 2018 Feb 15;13(2):e0191747. doi: 10.1371/journal.pone.0191747 (PMC5813932; doi:10.1371/journal.pone.0191747)
Supplement: S2 Table — Note: Measurement error is not presented as one of the measurement properties because it has not been evaluated in any of the articles. M = result of the methodological quality appraisal with a score on the 4-point rating scale based on the COSMIN: poor, fair, good, excellent R = result of the quality of measurement property appraisal with three possible categories: + = positive,? = inconclusive,— = negative; n.i. = not investigated, n.a. = not applicable, m = missing, CFA = confirmative factor analysis **Reference [43] also presents results of the development and validation for the SMDMQ (Taiwanese), however the results seem the exact same as presented in[42]; reference [43] was therefore left out in the data extraction and analysis and also not included in the number of included articles. ** The negative score is based on hypotheses that were not confirmed because correlations were high instead of medium-sized, thus, hypotheses testing actually showed that there is a strong relationship with instruments measuring the same construct. (DOCX) [file pone.0191747.s002.docx]

**S2 Table.** Methodological quality and quality of measurement properties of each article per measurement property and instrument version

| **Instrument** | **1^st^ author, year** | **Internal consistency** | | **Test-retest reliability** | | **Inter- rater reliability** | | **Intra-rater reliability** | | **Content validity** | | **Structural validity/ Item response theory (IRT)** | | **Hypotheses testing** | | **Cross-cultural validity (G) /Criterion validity (H)/ Responsiveness (I)** | |
| --- | --- | --- | --- | --- | --- | --- | --- | --- | --- | --- | --- | --- | --- | --- | --- | --- | --- |
|  |  | **M** | **R** | **M** | **R** | **M** | **R** | **M** | **R** | **M** | **R** | **M** | **R** | **M** | **R** | **M** | **R** |
| **Patient questionnaires** |  |  |  |  |  |  |  |  |  |  |  |  |  |  |  |  |  |
| PPC | Entwistle, 2001 [66] | n.a. |  | n.i. |  | n.a. |  | n.a. |  | n.i. |  | n.a. |  | poor | - |  |  |
| CPSpost | Entwistle, 2001 [66] | n.a. |  | n.i. |  | n.a. |  | n.a. |  | n.i. |  | n.a. |  | poor | - |  |  |
|  | Kremer, 2008 [67] | n.a. |  | n.i. |  | n.a. |  | n.a. |  | n.i. |  | n.a. |  | good | + |  |  |
| FPI | Martin, 2001 [28] | good | + | fair | + | n.a. |  | n.a. |  | n.i. |  | good | + | fair | - |  |  |
| COMRADE | Edwards, 2003 [38] | n.i. |  | n.i. |  | n.a. |  | n.a. |  | n.i. |  | fair | + | poor | - |  |  |
|  | Knapp, 2009 [68] | poor | + | n.i. |  | n.a. |  | n.a. |  | n.i. |  | good | - | fair | - |  |  |
| SDM-Q | Simon, 2006 [39] | n.a. |  | n.i. |  | n.a. |  | n.a. |  | n.i. |  | IRT: good | - | poor | - |  |  |
| SDM-Q-9 | Kriston, 2010 [29] | excellent | + | n.i. |  | n.a. |  | n.a. |  | n.i. |  | excellent | + | n.i. |  |  |  |
|  | Scholl, 2012 [69] | good | + | n.i. |  | n.a. |  | n.a. |  | n.i. |  | n.i. |  | good | - |  |  |
| SDM-Q-9 (Spanish) | De las Cuevas, 2014 [32] | poor | + | n.i. |  | n.a. |  | n.a. |  | poor | ? | good | + | n.i. |  | CC: fair | + |
|  | Alvarez, 2016 [30] | good | + | n.i. |  | n.i. |  | n.i. |  | n.i. |  | n.i. |  | n.i. |  | CC: poor | + |
|  | Ballesteros, 2017 [31] | good | + | n.i. |  | n.i. |  | n.i. |  | n.i. |  | CFA: good / IRT: good | CFA: +/ IRT: + | n.i. |  |  |  |
| SDM-Q-9 (Dutch) | Rodenburg-VandenBussche, 2015 [33] | excellent | + | n.i. |  | n.a. |  | n.a. |  | n.i. |  | excellent | + | fair | - |  |  |
| SDM-Q-9 PSY (Hebrew) | Zisman-Ilani, 2016 [34] | good | + | n.i. |  | n.a. |  | n.a. |  | n.i. |  | good | + | fair | + |  |  |
| SDM-Q-9 (English) | Alvarez, 2016 [30] | excellent | + | n.i. |  | n.i. |  | n.i. |  | n.i. |  | n.i. |  | n.i. |  | CC: poor | + |
| CollaboRATE | Barr, 2014 [70] | n.a. |  | n.i. |  | n.a. |  | good | + | n.i. |  | n.a. |  | fair | - | R: poor | ? |
| CollaboRATE (Swedish) | Rosenberg, 2017 [41] | poor | + | fair | - | n.i. |  | n.i. |  | n.i. |  | n.i. |  | fair | - |  |  |
| SMDMQ (Tawanese) | Chang, 2014 [42, 43] ** | n.a. |  | n.i. |  | n.a. |  | n.a. |  | poor | ? | IRT: good | - | n.i. |  |  |  |
| SDM Process Score | Fowler, in progress [44] | n.i. |  | n.i. |  | n.a. |  | n.a. |  | n.i. |  | n.i. |  | fair | + |  |  |
| MADM | Vedam, 2017 [45] | excellent | + | n.i. |  | n.i. |  | n.i. |  | n.i. |  | excellent | ? | poor | ? |  |  |
| Dyadic Option patient version (Swedish) | Rosenberg, 2017 [41] | poor | + | fair | - | n.i. |  | n.i. |  | n.i. |  | n.i. |  | fair | - |  |  |
| **Provider questionnaires** |  |  |  |  |  |  |  |  |  |  |  |  |  |  |  |  |  |
| SDM-Q-Doc | Scholl, 2012 [35] | excellent | + | n.i. |  | n.a. |  | n.a. |  | n.i. |  | excellent | + | n.i. |  |  |  |
| SDM-Q-DOC (Persian) | Ebrahimi, 2014 [46] | poor | + | poor | - | n.a. |  | n.a. |  | n.i. |  | n.i. |  | n.i. |  |  |  |
| SDM-Q-Doc (Dutch) | Rodenburg-VandenBussche, 2015 [33] | excellent | + | n.i. |  | n.a. |  | n.a. |  | n.i. |  | excellent | + | fair | - |  |  |
| SDM-Q-Doc (Spanish) | Calderon, 2017 [47] | poor | ? | n.i. |  | n.i. |  | n.i. |  | poor | ? | good | ? | poor | ? |  |  |
| **Observer-based coding schemes** |  |  |  |  |  |  |  |  |  |  |  |  |  |  |  |  |  |
| IDM | Weiss, 2008 [71] | n.i. |  | n.a. |  | n.i. |  | n.i. |  | n.i. |  | n.i. |  | fair | - |  |  |
| DSAT | Guimond, 2003 [49] | n.i. |  | n.a. |  | poor | - | n.i. |  | n.i. |  | n.i. |  | fair | - |  |  |
|  | Butow, 2010 [72] | n.i. |  | n.a. |  | n.i. |  | n.i. |  | n.i. |  | n.i. |  | fair | + |  |  |
| DSAT-10 | Stacey, 2008 [50] | n.i. |  | n.a. |  | good | - | n.i. |  | n.i. |  | n.i. |  | n.i. |  |  |  |
| OPTION | Elwyn, 2003 [51] | poor | + | n.a. |  | good | - | poor | - | n.i. |  | good | - | good | - |  |  |
|  | Butow, 2010 [72] | n.i. |  | n.a. |  | n.i. |  | n.i. |  | n.i. |  | n.i. |  | fair | + |  |  |
| OPTION (revised) | Elwyn, 2005 [52] | poor | - | n.a. |  | good | - | poor | - | n.i. |  | excellent | - | n.i. |  |  |  |
|  | Weiss, 2008 [71] | n.i. |  | n.a. |  | n.i. |  | n.i. |  | n.i. |  | n.i. |  | poor | - |  |  |
|  | Kasper, 2011 [73] | n.i. |  | n.a. |  | poor | + | poor | + | n.i. |  | n.i. |  | fair | - |  |  |
|  | Vortel, 2016 [74] | n.i. |  | n.a. |  | poor | - | n.i. |  | n.i. |  | n.i. |  | n.i. |  |  |  |
| OPTION (Italian) | Goss, 2007 [53] | fair | + | n.a. |  | fair | + | fair | + | n.i. |  | poor | ? | n.i. |  |  |  |
| OPTION (revised) (German) | Hirsch, 2011 [54] | poor | + | n.a. |  | fair | - | n.i. |  | n.i. |  | good | + | fair | - |  |  |
| OPTION (revised and modified) (German) | Keller, 2013 [55] | poor | + | n.a. |  | poor | - | n.i. |  | n.i. |  | n.i. |  | poor | - |  |  |
| OPTION^12^ (Dutch) | Stubenrouch, 2016 [56] | n.i. |  | n.a. |  | poor | - | n.i. |  | n.i. |  | n.i. |  | n.i. |  |  |  |
| OPTION^5 item^ | Barr, 2015 [75] | n.a. |  | n.a. |  | good | - | poor | + | n.i. |  | n.a. |  | good | + |  |  |
|  | Vortel, 2016 [74] | n.a. |  | n.a. |  | poor | + | n.i. |  | n.i. |  | n.a. |  | poor | + |  |  |
| OPTION^5^ (Dutch) | Stubenrouch, 2016 [56] | n.a. |  | n.a. |  | poor | - | n.i. |  | n.i. |  | n.a. |  | fair | + |  |  |
| RPAD | Shields, 2005 [58] | n.i. |  | n.a. |  | m | m | m | m | n.i. |  | n.i. |  | fair | - |  |  |
| DAS-O | Brown, 2011 [59] | n.i. |  | n.a. |  | poor | - | poor | - | good | + | n.i. |  | fair | -** |  |  |
| SDM Scale | Singh, 2010 [60] | poor | - | n.a. |  | poor | ? | poor | ? | n.i. |  | poor | - | fair | + |  |  |
| PES | Kearny, 2011 [61] | n.i. |  | n.a. |  | n.i. |  | n.i. |  | n.i. |  | n.i. |  | n.i. |  |  |  |
| DEEP-SDM | Clayman, 2012 [62] | n.i. |  | n.a. |  | n.i. |  | n.i. |  | n.i. |  | n.i. |  | n.i. |  |  |  |
| Shared decision making rating | Slayers, 2012 [63] | n.i. |  | n.a. |  | poor | + | n.i. |  | n.i. |  | n.i. |  | n.i. |  |  |  |
| MAPPIN'SDM_norge_ | Kienlin, 2016 [64] | n.i. |  | n.a. |  | fair | - | n.i. |  | n.i. |  | n.i. |  | fair | - | CV: poor | - |
| **Mixed instruments** |  |  |  |  |  |  |  |  |  |  |  |  |  |  |  |  |  |
| Dyadic OPTION^Patient^ | Melbourne, 2011 [26] | n.i. |  | n.i. |  | n.a. |  | n.a. |  | n.i. |  | n.i. |  | fair | - |  |  |
| Dyadic OPTION^Clinician^ | Melbourne, 2011 [26] | n.i. |  | n.i. |  | n.a. |  | n.a. |  | n.i. |  | n.i. |  | fair | + |  |  |
| MAPPIN'SDM patient questionnaire | Kasper, 2012 [27] | poor | + | n.i. |  | n.a. |  | n.a. |  | good | + | n.i. |  | fair | - |  |  |
| MAPPIN'SDM doctor questionnaire | Kasper, 2012 [27] | poor | + | n.i. |  | n.a. |  | n.a. |  | good | + | n.i. |  | fair | - |  |  |
| MAPPIN'SDM coding scheme | Kasper, 2012 [27] | n.i. |  | n.a. |  | fair | - | n.i. |  | n.i. |  | n.i. |  | fair | - |  |  |
|  | Kasper, 2012 [77] | n.i. |  | n.a. |  | good | - | n.i. |  | n.i. |  | n.i. |  | fair | - |  |  |

**Note:** Measurement error is not presented as one of the measurement properties because it has not been evaluated in any of the articles. M = result of the methodological quality appraisal with a score on the 4-point rating scale based on the COSMIN: poor, fair, good, excellent. R = result of the quality of measurement property appraisal with three possible categories: + = positive, ? = inconclusive, - = negative. n.i. = not investigated. n.a. = not applicable. m = missing. CFA= confirmative factor analysis.

*Reference [42] and reference [43] both presents results of the development and validation for the SMDMQ (Taiwanese), however the results presented seem the exact same in both articles, reference [43] was therefore left out in the data extraction and analysis and also not included in the number of included articles.

** The negative score is based on hypotheses that were not confirmed because correlations were high instead of medium-sized, thus, hypotheses testing actually showed that there is a strong relationship with instruments measuring the same construct.
